# Global nucleosome positioning regulates salicylic acid mediated transcription in *Arabidopsis thaliana*

**DOI:** 10.1186/s12870-014-0404-2

**Published:** 2015-01-21

**Authors:** Mala Singh, Sumit Kumar Bag, Archana Bhardwaj, Amol Ranjan, Shrikant Mantri, Deepti Nigam, Yogesh Kumar Sharma, Samir Vishwanath Sawant

**Affiliations:** CSIR-National Botanical Research Institute, Rana Pratap Marg, Lucknow, 226001 India; Academy of Scientific and Innovative Research (AcSIR), Anusandhan Bhawan, 2 Rafi Marg, New Delhi, 110 001 India; Department of Botany, Lucknow University, Lucknow, 226007 India

**Keywords:** Nucleosome, Plants, Chromatin, Salicylic Acid, *Arabidopsis thaliana*

## Abstract

**Background:**

The nucleosome positioning regulates the gene expression and many other DNA-related processes in eukaryotes. Genome-wide mapping of nucleosome positions and correlation of genome-wide nucleosomal remodeling with the changes in the gene expression can help us understanding gene regulation on genome level.

**Results:**

In the present study, we correlate the gene expression and the genomic nucleosomal remodeling in response to salicylic acid (SA) treatment in *A. thaliana.* We have mapped genome-wide nucleosomes by performing tiling microarray using 146 bp mononucleosomal template DNA. The average nucleosomal coverage is approximately 346 bp per nucleosome both under the control and the SA-treated conditions. The nucleosomal coverage is more in the coding region than in the 5′ regulatory regions. We observe approximately 50% nucleosomal remodeling on SA treatment where significant nucleosomal depletion and nucleosomal enrichment around the transcription start site (TSS) occur in SA induced genes and SA repressed genes respectively in response to SA treatment. Especially in the case of the SA-induced group, the nucleosomal remodeling over the minimal promoter in response to SA is especially significant in the Non-expresser of *PR1* (NPR1)-dependent genes. A detailed investigation of *npr1-1* mutant confirms a distinct role of NPR1 in the nucleosome remodeling over the core promoter. We have also identified several motifs for various hormonal responses; including ABRE elements in the remodeled nucleosomal regions around the promoter region in the SA regulated genes. We have further identified that the W-box and TGACG/C motif, reported to play an important role in SA-mediated induction, are enriched in nucleosome free regions (NFRs) of the promoter region of the SA induced genes.

**Conclusions:**

This is the first study reporting genome-wide effects of SA treatment on the chromatin architecture of *A. thaliana.* It also reports significant role of NPR1 in genome-wide nucleosomal remodeling in response to SA.

**Electronic supplementary material:**

The online version of this article (doi:10.1186/s12870-014-0404-2) contains supplementary material, which is available to authorized users.

## Background

It is now well known that the position of the nucleosome along a particular sequence of DNA has profound effects on its accessibility for all DNA processes such as transcription regulation, recombination, and repair [1-3]. For example, the nucleosome over the core promoter region of the pathogenesis-related gene (*PR-1a*) of tobacco is remodeled on induction with salicylic acid (SA) [4]. This suggests that the underlying DNA sequence and histone modifications play their role in gene activation, leading to the sliding of nucleosomes from the *PR-1a* core promoter. Several reports suggest that the position of the nucleosome is critical in globally regulating the *in vivo* binding of transcription factors by either allowing or blocking their binding to the nucleosome [5-9]. A nucleosome-depleted region (NDR) close to the transcription start site (TSS) is usually flanked by upstream and downstream positioned nucleosomes (denoted the −1 and +1 nucleosomes, respectively) that are often the starting points for the regular nucleosomal arrays. Protein factors along with the DNA and histone have a necessary role in nucleosome positioning [10-16]. In a previous study, five *in vivo* packing mechanisms of nucleosome organization have also been proposed for the nucleosomes in yeast [17].

Genome-wide mapping of nucleosome positions of eukaryotic genomes is a prerequisite to understand the basic mechanism of chromosomal organization. Previously genome-wide nucleosome positioning has been mapped and analyzed in *Saccharomyces cerevisiae, Drosophila melanogaster*, *Homo sapiens, Caenorhabditis elegans* and *A. thaliana* [15,18-27]. Different techniques have been used to assay nucleosome positioning in chromatin; for example, whole-genome tiling-microarray using mononucleosomal DNA [28], Micrococcal Nuclease (MNase1)-sequencing [22-25,27], which provides in-depth data on genome-wide nucleosomal positioning, etc.

Previously a study of global nucleosome positioning in *A. thaliana* has concluded for the first time that the nucleosomes are more densely packed in pericentromeric regions containing heavily methylated DNA than in the euchromatin region [27]. Taking into account the role of nucleosome in regulating eukaryotic transcription, it would be tempting to explore genome-wide nucleosomal dynamics in the context of transcriptional perturbation. Since plants are sessile organisms, they have developed intricate self defense mechanisms that enable them to cope with both biotic and abiotic stresses. The mechanism generally operates by generating the systemic acquired resistance (SAR) response mainly through SA-regulated and Jasmonic acid (JA)-regulated pathways with many other hormones [29,30]. This leads to massive changes in gene expression [31]. NPR1 has emerged as a key component which plays a significant role in regulation of these pathways and their cross-talk [32-34]. The molecule exists in the cytoplasm in multimeric form and is reduced to monomeric form to enter nucleus on being induced with SA. Overall, massive transcriptional reprogramming may be regulated at the chromatin level by nucleosomal remodeling. Thus, the present study aims at understanding the possible relationship between the dynamics of global nucleosomal positioning and SA-mediated transcriptional regulation in *A. thaliana*.

Briefly, we have mapped global nucleosome positioning in *A. thaliana* chromosomes under both control and SA-treated conditions. We have also correlated the changes in nucleosome positioning to the global transcriptional response to SA in *A. thaliana*. Our results report distinct chromatin remodeling in core promoter and 5′ upstream promoter regulatory regions of SA-induced genes. We also find a distinct role of non-expresser of PR1 (NPR1) in the nucleosomal remodeling in SA-regulated genes. For this, we identify SA- regulated NPR1-dependent (ND) and NPR1-independent (NI) genes by performing the whole genome expression analysis under control and SA-treated conditions both in the wild-type and the *npr1* mutant plants. The nucleosomal occupancy study of these groups reveals a correlation between SA-regulated ND genes and the dependence of NPR1 for nucleosomal remodeling at their core promoter region. We also find many defense related motifs both in the core promoter region and in the upstream regulatory region of SA regulated genes. While some of these are present in the nucleosome occupied regions (NORs), rest others are present in the nucleosome free region (NFRs).

## Methods

### Plant growth conditions and treatment with SA

Seed sterilization and plant growth conditions were followed as reported earlier [35] for both *A. thaliana* Col-0 (used as the wild-type) and *npr1-1* mutant. All seeds were grown *in vitro* in MS (Murashige and Skoog) medium by keeping them on a mesh for stratification at 4°C for 2 days before placing them in the growth chamber. *A. thaliana* plants were grown under controlled environmental conditions (22 ± 1°C, 120 μmol m^−2^ sec^−1^, and 16 h light/8 h dark cycle). After two weeks of growth, the medium was exchanged either with water or with 2 mM SA, followed by 24 h incubation in light at 22 ± 1°C.

### Nuclei isolation and digestion with MNase1

The nuclei were isolated as described [36], with slight modifications. Briefly, 10 g of seedlings (Col-0 or *npr1-1*) were treated either with water or SA, rinsed with water, blotted dry, and ground to powder in liquid nitrogen. Nuclei isolation buffer NIB1 (0.5 M hexylene glycol, 20 mM KCl, 20 mM PIPES (pH 6.5), 0.5 mM EDTA, 0.1% Triton X-100, and 7 mM 2-mercaptoethanol) was added to it. The extract was first filtered through a 4-layered muslin cloth and then sequentially through 80, 60, 40, and 20 μm mesh sieves. The filtrate was centrifuged at 3000 g at 4°C for 10 min. The pellet was suspended in NIB2 (NIB1 without Triton X-100) and centrifuged again. The pellet was suspended in 5% percoll (U.S. Biologicals), loaded on a 20–80% percoll step gradient, and centrifuged. The nuclei were removed from the 20–80% percoll interface, washed in NIB2, and resuspended in NIB1 buffer. The nuclear preparation was incubated with micrococcal nuclease (300 units/μl, Fermentas# EN0181) in a buffer containing 25 mM KCl, 4 mM MgCl_2_, 1 mM CaCl_2_, 50 mM Tris-Cl (pH 7.4), and 12.5% glycerol at 37°C for 10 min at the concentration of 1unit/μg of genomic DNA from untreated sample and 0.6unit/μg of genomic DNA from treated sample (see Additional file 1). The reaction was stopped by adding an equal volume of 2% SDS, 0.2 M NaCl, 10 mM EDTA, 10 mM EGTA, and 50 mM Tris-Cl (pH 8); treated with proteinase K (100 μg/ml, Ambion#2546) for 1 h at 55°C, then with RNase A (100 μg/ml, Qiagen#19101) at 37°C for 30 min, extracted by phenol: chloroform, and precipitated in ethanol. The DNA was separated on 1.5% agarose gel, and fragments of an average size of 150 bp were purified (Sigma#NA1020). The experiment was repeated three times each for control and SA-treated seedlings.

The control genomic DNA for the hybridization experiments was prepared from nuclear genomic DNA extraction and sonicated to obtain fragments of an approximate size of 200–500 bp. The experiment was repeated two times.

### Tiling array experiment

Isolated mononucleosomal DNA fragments (SA-treated and untreated) and genomic control DNA were amplified with random primers (N6), Klenow fragment (3′ → 5′ exo^−^; NEB#M0210L) and 25 mM dNTP (with dTTP and dUTP in 4:1 ratio) in 20 μl aliquots for a total volume of 100 μl that was sufficient to obtain approximately 10 μg of purified PCR products for each sample. The PCR products were purified with the PCR cleanup kit (Sigma, #NA1020). Fragmentation and labeling was done as per Affymetrix ChIP Assay Protocol (P/N 702238) using GeneChip® WT Double-Stranded DNA Terminal Labeling Kit (P/N 900812). Hybridization, washing, and staining were done according to the Affymetrix Chromatin Immunoprecipitation Assay Protocol onto GeneChip® *A. thaliana* Tiling 1.0R array (Reverse) using GeneChip® Hybridization, Wash, and Stain Kit (P/N 900720) on Affymetrix Fluidics station 450 according to the manufacturer’s protocol. Arrays were scanned on an Affymetrix GeneChip® 3000 7G scanner, and the signals were quantified with the Affymetrix GeneChip® Operating Software (GCOS) to generate *cel* files. The data can be found in NCBI public repository as GSE25553 [37]. The experiments were performed in similar way for all the eight templates (three each for control and SA-treated and two for the genomic DNA).

### Processing of tiling array data

In the subsequent analysis, raw data from all probes were mapped onto TAIR10. Tiling Analysis software (TAS) from Affymetrix technology was used to analyze tiling array data (*cel* files), which included the use of quantile normalization, probe analysis, and two-sample analysis to generate normalized log2 transformed values. The two-sample comparison analysis included datasets, either control versus genomic or SA-treated versus genomic, that could help in identifying consistently differentially enriched regions along the genome in control and SA-treated conditions respectively as compared to background region. Since genomic DNA was randomly sheared DNA, the comparison also yielded negative log2 transformed signal intensity values for certain probe sets in both cases. Throughout this article, this log2 transformed signal intensity value has been directly referred to as *enrichment value*. These enrichment values were then used for further analysis in a comparative fashion in order to determine the nucleosomal remodeling across the genome.

### Definition of stringency level

Seven different enrichment values (≥0.0, ≥−0.01, ≥−0.1, ≥−0.5, ≥−1.0, ≥−1.5 and ≥−2.0) have been used as seven stringency levels. For example, stringency level ≥0.0 means that if a probe has enrichment value ≥0.0, then only it may serve as a nucleosomal signal, not otherwise.

### Development of custom scripts

Four different custom scripts were developed to predict nucleosomal occupied regions from the signal intensity data of TAS (Tilling Array analysis) in different steps –

1. C script to develop the dataset of distinct and fuzzy nucleosomes: Tab separated file containing the genomic positions and the log2 transformed signal intensity values from all the three replicates was used as input for both control and SA treated datasets to get the nucleosomal regions (distinct and fuzzy) at different stringency levels. For a given stringency level, the presence of a nucleosome is inferred, if the enrichment value is ≥ the value of the stringency level continuously at 4 probes (for distinct nucleosomes) or at >4 probes (for fuzzy nucleosomes) in all the three replicates in the dataset under study. For example, for inferring the presence of a distinct nucleosome at stringency level ≥0.0, four continuous probes should have enrichment value ≥0.0.
2. Perl script to develop a database of global positions of TSS, start codon, stop codon, and mRNA end for all the genes along all the five chromosomes of *A. thaliana* from TAIR10 tabular files.
3. C script to find out the remodeled nucleosomal regions and common nucleosomal regions: The nucleosomal regions present either only in control conditions or only in SA treated conditions were considered as remodeled nucleosomal regions and the nucleosomal regions present in both conditions were considered as the common nucleosomal regions for the purpose of input data in this script.
4. C script to search every 50 bp window size for the nucleosomal coverage of 1000 bp upstream and downstream from TSS site of the genes in different groups: Two different input files were used –
  1. The strand position (either forward or reverse) and the TSS (Transcription start site) of gene.
  2. The nucleosomal dataset both for distinct and fuzzy nucleosomes (coming from first script).

If a continuous stretch of ≥25 bp was predicted to be having nucleosome occupancy in 50 bp window, then it was marked as count 1 (considered as having a nucleosome). On the other hand, if the continuous nucleosome coverage was <25 bp in 50 bp window, then it was marked as count 0 (considered as having no nucleosome).

All of these scripts are available at http://ncgd.nbri.res.in/nucleosome_prediction.aspx.

### Identification of genome-wide nucleosome regions in control and SA-treated conditions

A total of 140 datasets were developed over seven stringency levels, two conditions and two types (distinct and fuzzy) of nucleosomes for the five chromosomes using first custom script. Nucleosome regions covering an area of 131–161 bp represented distinct nucleosomes and the nucleosome regions covering an area ≥161 bp represented fuzzy nucleosomes.

### Calculation of nucleosomal coverage over different parts of gene

The tiling array data were mapped for all the five chromosomes, and the nucleosome occupancy was calculated in different parts of the genes identified using second custom script. The nucleosomal coverage was calculated at stringency ≥−1.0 as size of the chromosome (in bp) divided by the total region covered by nucleosomes (distinct + fuzzy) for all the five chromosomes of *A. thaliana* (see Additional file 2).

### Identification of remodeled nucleosomes on SA treatment

Nucleosomal regions were identified for the following conditions using third custom script

- Regions having nucleosomes shifted/removed under SA-treated condition as compared to control condition.
- Regions having nucleosomes added under SA-treated condition as compared to control condition.
- Regions retaining nucleosomes under both conditions.

The first two regions taken together (i.e. a + b) formed a remodeled nucleosomal region, whereas all regions taken together formed total nucleosomal coverage (i.e. a + b + c).

### RNA extraction

Total RNA was isolated from two week old seedlings (both Col-0 and *npr1-1*) in two independent biological replicates using Spectrum plant total RNA Kit (Sigma-Aldrich) according to the manufacturer’s instructions and eluted with nuclease-free water. After DNaseI (Ambion) treatment, the RNA was quantified and checked for integrity using a Bioanalyzer (Agilent, Inc., Palo Alto, CA, USA).

### RNA amplification and labeling

Direct labeling procedure was used i.e., double-stranded cDNA was synthesized using 250 ng total RNA, either with an oligo-dT primer (for QPCR purpose) or a T7 promoter-containing oligo (dT) primer, followed by *in vitro* transcription and labeling using a GeneChip® 3′ IVT Express kit (Affymetrix) to generate cRNA as per manufacturer’s protocol.

### Microarray hybridization

The cRNA was fragmented to approx. <100 bases, hybridized to ATH1 Genome chip and were incubated at 45°C temperature for 16 hrs at 60 rpm in hybridization chamber. Washing and staining was performed by using an Affymetrix Fluidics Station 450 according to the manufacturer’s instructions (GeneChip expression analysis technical manual). The arrays were scanned using Gene Chip Scanner 3000 7G (Affymetrix). Scanned images were processed and analyzed using GCOS software (Affymetrix). The data has been deposited in NCBI (GSE 51626).

### Image acquisition and statistical analysis of salicylic acid treated oligonucleotide microarray data

Arrays Images were captured and then raw cel files were analyzed using affylmGUI biocondcutor R package for Robust Multi-array Average (RMA) algorithm [38] by performing model-based background correction, normalization and summarization across the probe level. Identification of statistically significant (P-values ≤0.05) differentially expressed genes as determined by affylmGUI biocondcutor R package was done using the fold change criteria (see Additional file 1).

### Identification of SA induced (SI), SA repressed (SR) and uninduced genes

The fold change criteria along with P-values ≤0.05 has been used to classify the genes in different groups.

1. Genes with log2 fold change ≥2 were considered as up-regulated, hence were called as SI genes,
2. Genes with fold change ≤−2 were considered as down-regulated, hence were called as SR group,
3. Uninduced genes with an equal and consistent expression profile in all the replicates of SA and control array files were grouped into silent (SIL) genes (expression values ≤0) and constitutive (CON) genes (expression values ≥3).

### Identification of ND and NI genes

Expression profiling experiments were performed for both wild-type and *npr1-1* mutants under untreated and treated conditions (GSE51626) for the identification of ND and NI genes. Thereafter, two-way ANOVA was used under R script developed by Pavlidis *et al.* [39] for selecting statistically significant SA-regulated ND and SA-regulated NI genes. Normalized log2 transformed values of all replicates of untreated and treated Col-0 and *npr1-1* mutant array files were processed for ANOVA analysis. Genes with adjusted P-value ≤0.05 were considered as SA regulated genes (as performed by Q-value [40] package implemented in R). For SA-regulated ND genes, the genes with P-value ≤0.0001 (for NPR1-dependence) were selected while for SA-regulated NI genes P-value ≥0.1 were selected.

Further, SA-regulated ND genes were classified into two groups i.e. SI-ND and SR-ND genes based on the fold change value of the genes upon SA treatment in wild-type plants. Similarly, SA-regulated NI genes were classified into two groups i.e. SI-NI and SR-NI genes.

### Quantitative PCR for the analysis of nucleosomal occupancy and transcript abundance

For validating the ≥−1.0 stringency level, primers were designed both from the regions showing the presence of the nucleosome in both control and SA-treated conditions (exonic regions) and from the regions showing the absence of the nucleosome in both control and SA-treated conditions (intergenic-UTR region for At1g53790, exon-UTR region for At2g28650, intergenic region for At3g55430 and the exonic region for At4g07408), as depicted in our tiling array analysis (see Additional files 1 and 3) using Primer Express software (Applied Biosystems). The PCR efficiency of all the primers was checked by using the *A. thaliana* genomic DNA as a template. QPCR was carried out using ABI Taqman chemistry with untreated mononucleosomal DNA template on ABI Prism 7000 sequence detection system (Applied Biosystems). The Ct (threshold cycle) value for each gene was determined and QPCR data analysis was done by comparing Ct values [41,42]. The experiments were repeated twice with at least 3 biological replicates.

To assess the effect of NPR1 on nucleosomal occupancy on the promoter region in all the six groups, mononucleosomes were prepared as described earlier from both treated and untreated wild-type and *npr1-1* mutant seedlings. Next, QPCR was performed for the assessment of nucleosomal occupancy at the promoter loci of three representative genes for each class (total six classes; i.e. SI-ND genes– AT1G28480, AT1G80590, AT3G56400; SR-ND genes– AT5G38700, AT4G37800, AT5G13220; SI-NI genes– AT1G05700, AT1G09380, AT1G68450; SR-NI genes– AT4G21850, AT1G66100, AT4G34410; CON genes – AT5G02380, AT5G02500, AT5G60390 and SIL genes – AT1G27380, AT4G23780, AT3G26130). For the internal control set, five exonic regions (AT4G04570, AT5G64905, AT1G24020, AT4G29030, and AT1G14150) were selected; the selected exons did not show nucleosomal remodeling on SA treatment. Moreover, they did not belong to any groups such as SI, SR, ND, or NI. The average C_t_ values of each of the three genes was normalized to the average C_t_ values of a complete set of internal control genes to obtain ΔC_t_ values. Similar treatment was followed for all the sets; that is, wild-type Control (Wt-Ct), wild-type SA treated (Wt-SA), *npr1-1* Control (NPR1-Ct), and *npr1-1* SA treated (NPR1-SA). Thereafter, the average ΔC_t_ value for the set of three genes was determined and average fold change was calculated and plotted along with standard error (SE). Student’s *t*-test was applied to test the significance of the changes under both conditions in wild-type versus mutant plants in a pair-wise manner.

Analysis of the relative transcript abundance of each group (for the same genes used for the nucleosomal occupancy analysis in different groups) was done using total RNA for each group prepared under similar conditions and using the same analyses strategy as done for studying nucleosomal occupancy described earlier.

### Mapping of the nucleosomal coverage

Only the regions consisting of −1000 bp to +1000 bp with respect to TSS of a particular gene were considered for this purpose. Overlapping coordinates or genomic regions were filtered from −1000 to +1000 bp region with respect to TSS in all genes in the constitutive (CON), silent (SIL), SA induced (SI), SA repressed (SR), SA induced-ND (SI-ND), SA repressed-ND (SR-ND), SA induced-NI (SI-NI), and SA repressed-NI (SR-NI) groups. These regions were then removed from each group. The regions were mapped for the nucleosomal coverage (at stringency ≥−1.0 as earlier calculated using custom script 1) using fourth custom script. Statistical significance analysis at a P-value ≤0.05 was performed using Student’s *t*-test (N = 3, null hypothesis of no significance difference) for each window in all the groups. Only the adjusted P-values ≤0.05 (calculated using Q-value package) were considered as significant.

### Calculation of nucleosomal remodeling

The presence or absence of a nucleosome in −1000 to +1000 bp-long region was calculated on per gene basis for all genes for both control and SA-treated conditions in different groups (CON, SIL, SI and SR, SI-ND, SR-ND, SI-NI and SR-NI). Based on the remodeling at each position, percentage of the genes showing remodeling was calculated for each position for all groups. A graph representing the percentage of genes having nucleosome at each position was drawn along with the statistical significance data for comparison between different groups. Statistical analysis was done for −1000 to +1000 bp region with respect to TSS over a window of 150 bp (so as to cover one nucleosome long region) to find differences in the form of nucleosomal enrichment and nucleosomal depletion at the proximal promoter region among SI versus unaffected groups (i.e. CON and SIL groups together) and SR versus unaffected groups, SI-ND versus SI-NI and SR-ND versus SR-NI. For this, Student’s *t*-test (N = 3, null hypothesis of no significance difference) was applied along with the Q-value package for multiple hypothesis testing correction. Only the adjusted P-values ≤0.05 were considered as significant.

### Gene Ontology analysis

All the groups were analyzed for their enriched gene ontologies using agriGO software [43] under SEA (singular enrichment analysis) using Fischer statistical test under adjusted P-values ≤0.05 using Yekutieli method.

### Prediction of nucleosomal occupied, nucleosomal free and remodeled nucleosomal regions

Third custom script was used to count the number of nucleosomal occupied, nucleosomal free and remodeled nucleosomal regions in the dataset of different groups. Nucleosomal bases were calculated in control as well as SA treated condition. Common nucleosomal bases present in the both the conditions were also calculated. Remodeled nucleosomal bases were calculated by subtracting the common nucleosomal bases from total nucleosomal bases (Control and SA treated).

### Motif finding methods

a) For SI genes: Remodeled nucleosomal regions (199) (Total bp = 28360) of 1000 bp upstream regions from SI genes (111) were extracted from TAIR10 Database. Motif analysis was done for these regions. MEME (ver. 4.2) [44] was deployed using ZOOPS model (with minimum motif width = 6 bases and maximum motif width = 10 bases and maximum number of motifs to return = 10) to these regions. It identified several interesting motifs which were annotated with PLACE [45] database.

B) For SR genes: The promoter regions −600 to −800 upstream TSS (200 bp) of SI and SR genes were extracted from TAIR10 database. Additionally, as control dataset, the remodeled nucleosomal regions (191695 regions; Total bp = 32052083) from whole genome of *A. Thaliana* (W) were used to identify novel cis-regulatory elements specifically enriched in SR genes (with respect to SI genes) present in the remodeled regions. A database was constructed consisting of all possible combinations of 6 bases length. Thereafter, the motif analysis was done using following two methods:

i) *Lift method*
  Lift [46] was calculated for the database containing all the possible combinations in SI versus W and SR versus W. Finally, the ratio of both was calculated to find out the enriched motif of SR with respect to SI.
ii) *Motif enrichment (OE) method*
  The expected occurrence of a particular hexameric motif was counted by the method used for counting W-Box and TGA motifs. Thereafter, the ratios between observed and expected occurrence for each motif found in the SI and W remodeled regions were calculated. Similarly, the ratios between observed and expected occurrence for each motif found in the SR and W remodeled regions was also calculated. Finally, ratio of motifs enriched in SR as compared to W and the motif enriched in SI as compared to W was calculated to obtain enriched motif in SR genes with respect to SI genes.

### Nucleosome coverage on W box and TGA motifs

Nucleosomal regions from the 1000 bp upstream TSS (promoter regions) were extracted for all the groups. In these regions, the enrichment of *cis*-regulatory W-box motif (TTGAC) and TGA motif (TGACG/C) was independently calculated for the following four regions:

- total promoter region,
- region having the nucleosome occupied under both conditions (NOR),
- region lacking the nucleosome under any of the two conditions ( NFR), and
- regions with remodeled nucleosomes.

First of all, the local nucleotide frequency (F) of a base was calculated in the above mentioned regions as per the formula –

Say, T = total no of all bases in a particular region

Then F = (count of the a particular base in a region)/T

Thereafter, the expected frequency of occurrence of a particular *cis*-regulatory motif was calculated based on the local nucleotide frequency of each constituent base of the motif under study using the following formula:

$$ E=\frac{{\displaystyle {\prod}_1^NF}}{N}\ast T $$

E = Expected occurrence of the motif, N = Number of the constituent bases in the motif.

The occurrence of these motifs has been further corrected for their expected frequency of occurrences and for the differences due to database sizes for total number of promoters, NORs, remodeled nucleosomes, and NFRs. The corrected frequency of occurrence of a particular motif was calculated by normalizing the observed frequency with the expected frequency of their occurrence. Next, graphs showing the significance of each motif in all groups were plotted at 95% binomial confidence interval with respect to the total promoter region.

## Results and discussion

### Global mapping of nucleosome positioning on the *A. thaliana* genome

**Figure 1:**
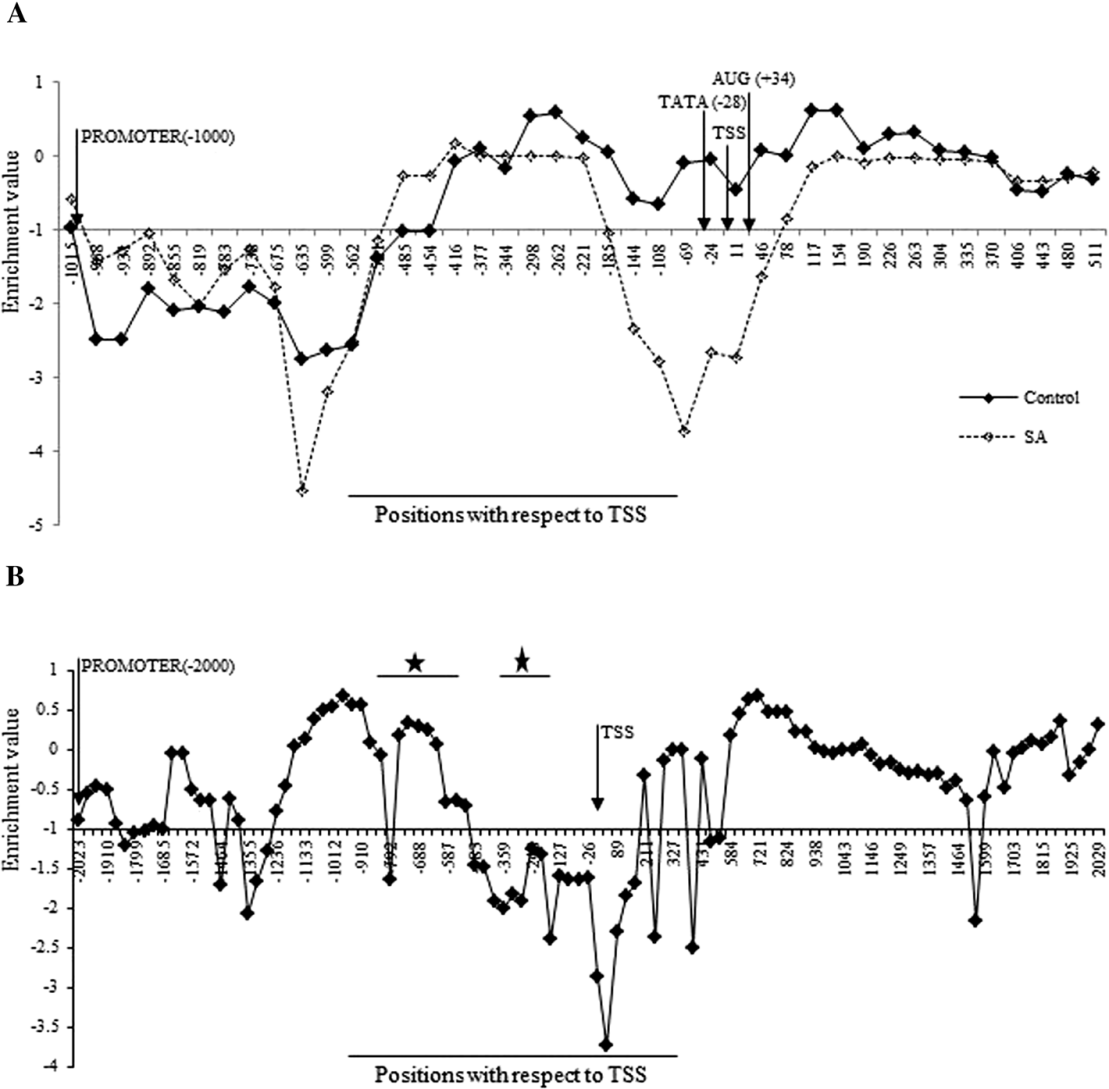
***Graph showing nucleosome positioning at At2g14610 and At1g77120 loci to determine stringency criteria.*** **A**– depicts the nucleosomal remodeling around At2g14610 promoter regulatory region. The X-axis denotes the position with respect to Transcription start site (TSS) on chromosome 2 and Y-axis denotes the enrichment values. **B**– Validation of nucleosomal stringency level by mapping on Adh gene (AT1G77120, previously mapped). The X-axis denotes the position with respect to TSS on chromosome 1 and Y-axis denotes the enrichment values. The asterisk shows the regions having nucleosome according to the previous mapping done by DNase assay (Vega-Palas and Ferl, 1995).

The MNase1 digestion strategy is widely used to map nucleosome position and occupancy, based on its ability to produce sequence-dependent cleavages in DNA preferentially within linker regions. An earlier publication [47] claims that the mapping of nucleosome positioning using MNase1 generates a sequence-dependent bias in the resulting fragments, which may affect sequence-dependent models for nucleosome positioning. Still, it is unlikely that such biasness is differentially influenced by SA treatment. Hence, it can be safely used to understand differential nucleosome positioning in *A. thaliana* on SA treatment. To determine the changes in the nucleosome positions that eventually lead to changes in the gene expression, 2-week old control or SA-treated *A. thaliana* seedlings were used for mononucleosomal DNA preparation (see Additional file 1). The mononucleosomal DNA was then hybridized to Affymetrix GeneChip® Arabidopsis Tiling 1.0R Array. Custom scripts were developed and used for assigning nucleosomes where nucleosomes were identified at different stringency levels (i.e. log2 transformed signal intensity levels termed as *enrichment values*, described in Methods). *PR1* (pathogenesis-related gene1) is a marker gene of the SA pathway. Earlier, it was demonstrated by us in case of *Nicotiana tabacum* that a distinct nucleosome spans the TATA-box and TSS of *PR-1a* minimal promoter at the resting state, which gets remodeled and removed on activation with SA [4]. *PR1* in *A. thaliana*, similar to tobacco *PR-1a*, is also an SA-induced NPR1-dependent gene; thus, *PR1* was selected to validate the results obtained by tiling microarray on SA-induced NPR1-dependent genes. The *A. thaliana PR1* gene (At2g14610) is located on the reverse strand of chromosome 2; its genic region spans from position 6,241,704 to 6,243,463 (1760 bp) encompassing the promoter region which spans from 6,242,463 to 6,243,463 with distinct TATA-box at position 6,242,491 (−28 bp with respect to TSS; Figure 1A). The nucleosome positioning around *PR1* gene in the tiling microarray data was examined. At stringency level ≥ −1.0 under control condition, nucleosome signals were observed spanning from 6,242,452 to 6,242,879; covering AUG, TSS, and TATA-box (−416 to +11 bp with respect to TSS); and extending further into both the promoter region and the region downstream TSS, indicating the presence of many nucleosomes in that region. After SA induction, distinct MNase1 sensitivity was observed in this region and, thus, lack of hybridization signals around the region −221 to +78 bp, indicating the nucleosomal remodeling and removal of the nucleosome from the region (Figure 1A). The nucleosomal coverage on *PR1* at −24 position showed a conspicuous decline from −0.04 (control) to −2.67 (SA; Figure 1A), reconfirming depletion of the nucleosome at the core promoter in SA-induced genes. Since the results were validated at the stringency level of ≥ −1.0 enrichment value by finding a nucleosome at the core promoter of the *PR1* gene, it was subjectively decided to use the stringency of ≥ −1.0 enrichment value for subsequent analysis.

This stringency level was cross-validated by mapping the nucleosome occupancy over −2000 to 2000 bp region with respect to TSS of Alcohol dehydrogenase1 gene (*Adh;* AT1G77120; Figure 1B). This region of the gene has earlier been mapped [48] and is shown to contain three nucleosomes in the promoter region (0 to −1000 bp with respect to TSS), where one nucleosome is loosely positioned at −130 to −350 bp with a best fit at −230 bp and other two nucleosomes span −500 to −800 bp. The downstream region is also occupied by clear nucleosome ladders. A clear nucleosome ladder was found both downstream and upstream TSS in the map generated by us, where the nucleosome occupied −800 to −495 bp region with respect to TSS. The only minor observable difference was that a loosely positioned nucleosome reported at −130 to −350 bp position could not be interpreted clearly at the stringency level of ≥−1.0 enrichment values in our data. However, if the stringency level is reduced from ≥−1.0 to ≥−2.0 in our data, even this nucleosome can also be identified. Thus, our results are in good agreement with an already published nucleosome map of *Adh* gene [48], thereby justifying the ≥−1.0 stringency criteria experimentally, to assign nucleosome positioning genome wide (Figure 1B).

The chosen stringency level was subjected to further verification using QPCR analysis over four loci having a nucleosome under control conditions and four other loci lacking any nucleosome under control conditions. The results are concurrent with the interpretation made for each gene locus based on the tilling microarray experiments (see Additional files 1, 3 and 4). Thus, in our analysis, the presence of four continuous probes, consistently in three independent experiments, with enrichment value ≥−1.0 marks a distinct nucleosome and likewise, the presence of more than four continuous probes marks a fuzzy nucleosome (See Methods). Our results show that fuzzy nucleosome signals are several folds higher than those of the distinct nucleosomes at the chosen stringency level. Additional file 5 shows the number of bases that covered as distinct nucleosomes and the number of bases covered as fuzzy nucleosomal regions identified at the different stringency levels (See Methods). The number of distinct and fuzzy nucleosomal regions obtained is almost consistent in all the chromosomes under both control and SA-treated conditions. The fuzzy nucleosomal regions were approximately four times higher than the distinct nucleosomes at the most stringent level (≥0) in terms of the number of nucleosomes (and 8-10 times higher in terms of number of bases covered) and more than ten-time higher at the least stringent level (≥−2.0) in terms of the number of nucleosomes (and approximately 60-110 time higher in terms of number of bases covered). At ≥−1.0 stringency level, the fuzzy nucleosomal regions are 8-10 folds higher than the distinct nucleosomes in terms of the number of nucleosomes (and 36-39 times higher in terms of number of bases covered). Below −1.0, the fuzzy nucleosomal regions extend so much that the coverage by distinct nucleosome (both in term of bases and in term of the number of nucleosomes) decreases. This extension also affects the number of fuzzy nucleosomes negatively. While the overall coverage as fuzzy region increases, the number of fuzzy nucleosome decreases, indicating that the fuzzy regions eventually merge to increase their span. In conclusion, the table shows the gregariousness of data expected if stringency level below ≥−1.0 were to be used for this study. The higher abundance of the fuzzy nucleosomal region can be partially attributed to the presence of more than one nucleosome that cannot be resolved by tiling microarray due to its limited resolution of 35 bp [49] and partially, to the use of complete *A. thaliana* seedlings (a composite tissue) for extracting mononucleosomes, thus interpreting only an average position of a nucleosome rather than the distinct nucleosome present in a specific cell lineage, as also observed in the case of *Caenorhabditis elegans* [25].

### Global nucleosomal coverage follows similar pattern across the genomes

The overall nucleosomal coverage obtained in our study is approximately 346 bp per nucleosome on all the chromosomes of *A. thaliana* (see Additional file 2). Thus the number of nucleosomes per 1000 bp in our study is much less than the number of nucleosomes per 1000 bp with the nucleosomal coverage of 211 bp per nucleosome obtained by Yuan *et al.* in yeast [15] and 200 bp per nucleosome obtained by Schones *et al.* in the human T4 cell line [23]. In addition, our study shows approximately 50% nucleosomal remodeling on SA treatment (see Additional file 6). This means that although the nucleosome coverage is approximately 42-44% under either of the two conditions and that approximately 56-58% region of the genome remains free under either condition, 56.34% of the genome is the NOR when both conditions are taken together. Approximately 50% of this NOR (26.9% of the total genome) either gains or loses the nucleosomes on SA treatment and hence is unique to each subset. Remaining approximately 50% NOR (29.44% out of the total genome) is common to both conditions. Further, the estimate of approximately 56-58% NFR may be an over estimation, considering the limitation of using GeneChip® Arabidopsis Tiling 1.0R Array, which covers not more than 88% of *A. thaliana* genome. Further, the nucleosomal remodeling post SA treatment does not show any bias for a specific chromosome (see Additional file 6).

Next, the nucleosomal coverage in non-coding regulatory regions, namely the promoter region, 5′UTR, and 3′UTR and the coding region was examined on each chromosome. The coding regions show a significantly higher nucleosomal percentage (more than 60%) than the regulatory region (around 40%, see Additional file 7). These observations are consistent with earlier studies in the case of *Saccharomyces cerevisae*, *Caenorhabditis elegans*, and *Homo sapiens* [15,23,25,50], where lower nucleosomal occupancy in the regulatory region has been reported.

**Figure 2:**
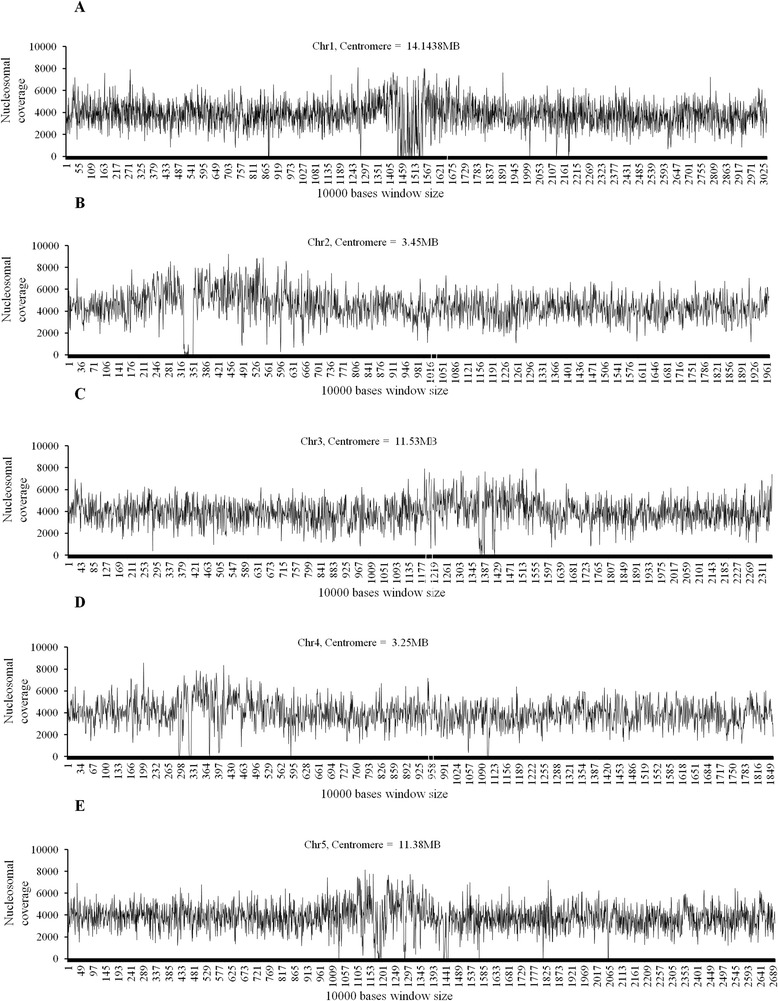
***Distribution of Nucleosome Coverage in the chromosomes.*** X axis **(A-E)** represents the 10000 bases window size in chromosomes 1–5 respectively and Y axis **(A-E)** represents the nucleosomal coverage in the window size of 10000 bases.

Nucleosomal coverage was also compared in pericentromeric and centromeric regions along the five chromosomes in *A. thaliana* by plotting the nucleosomal coverage across the 10000 bp window (Figure 2). It was found that the nucleosomal coverage is consistently higher over entire chromosome (marginally peaking at the pericentromeric regions of all chromosomes, being 4000-5000 bp per 10000 bp) as compared with the centromere with <1000 bp coverage (see Additional file 8). The depleted nucleosomal coverage over the centromeric region is solely because of poor probe density over the region. There is approximately equal distribution of probes throughout the genome except over the centromeric and pericentromeric regions which have rather thin density of probes. Additionally, the nucleosomal coverage is directly correlated to the probe density over both the centromeric and pericentromeric regions, except for chromosome 2 (see Additional file 8, correlation data). These results agree with earlier reports in which the authors have found high content of nucleosome in pericentromeric heterochromatin regions arranged as a regularly spaced array [27].

### Genome-wide changes occur in nucleosome positioning in response to SA

**Figure 3:**
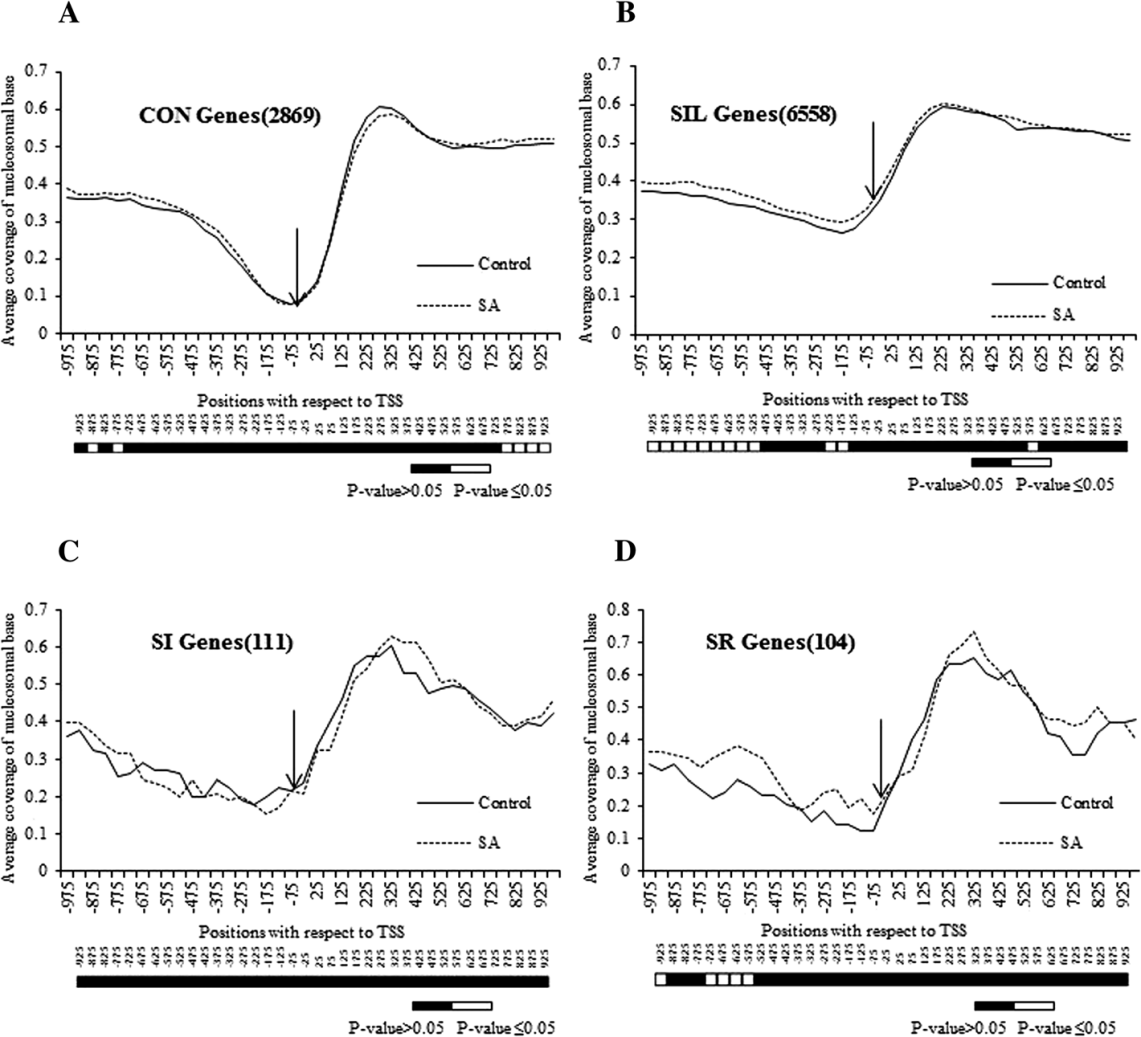
***Nucleosomal occupancy is always depleted at Transcription start site (TSS).*** Coverage of -1000 bp to +1000 bp long region by nucleosome was done for different groups of genes (**A** – constitutive-CON; **B** – silent-SIL; **C** – SA induced-SI and **D** – SA repressed-SR). X-axis represents the positions with respect to TSS (where −75 means −51 to −100) and Y-axis represents average coverage of nucleosomal base in each figure. The numbers of genes taken in each group is given in parentheses. The −25 position is shown with arrow. Bar below each figure shows the comparative statistical significance difference for each window.

To understand the genome-wide nucleosomal remodeling in response to SA in *A. thaliana*, global gene expression profiling was examined using *A. thaliana* ATH1 chip (GSE51626) in conjunction to our nucleosomal mapping experiment. SI, SR, CON and SIL groups were identified (see Additional file 9). Average nucleosome coverage around the −1000 to +1000 bp regions with respect to TSS was examined in the genes of the groups specified earlier in a window of 50 bp. All the groups show considerably higher nucleosomal coverage in the coding region ranging from 0.6 to 0.7 as compared to the promoter region, where it ranges from 0.1 to 0.4 (Figure 3A-D).

Another distinct feature commonly observed in all classes is the presence of a nucleosomal valley around the minimal promoter region within -200 bp upstream of TSS (Figure 3). This is consistent with the previous findings in other studies [15,17,51], where also the TSS has been shown to be free of the nucleosome. In *Saccharomyces cerevisae*, the authors have consistently found NFRs in the promoter region [15,17]. Likewise, the promoters of actively expressing genes in *Homo sapiens* also possess nucleosome-free TSS flanked by positioned nucleosomes [51]. Thus, the average low nucleosomal coverage at TSS seems to be a ubiquitous property of all the genes across the genomes. In our study, the average coverage at this valley always remains below 0.23 (Figure 3A-D) in all groups (reaching as low as 0.09 in the CON group), except the SIL group where it is above 0.35 (Figure 3B). For this group, the valley is considerably shallower than others, indicating a dense chromatin structure around the minimal promoter region. A possible reason accounting for this shallowness may be that since these genes are not expressed, they maintain the presence of nucleosomes at their minimal promoter region. It should be noted that the average nucleosomal occupancy at the −25 window also remains nearly insensitive to SA treatment in all groups.

**Figure 4:**
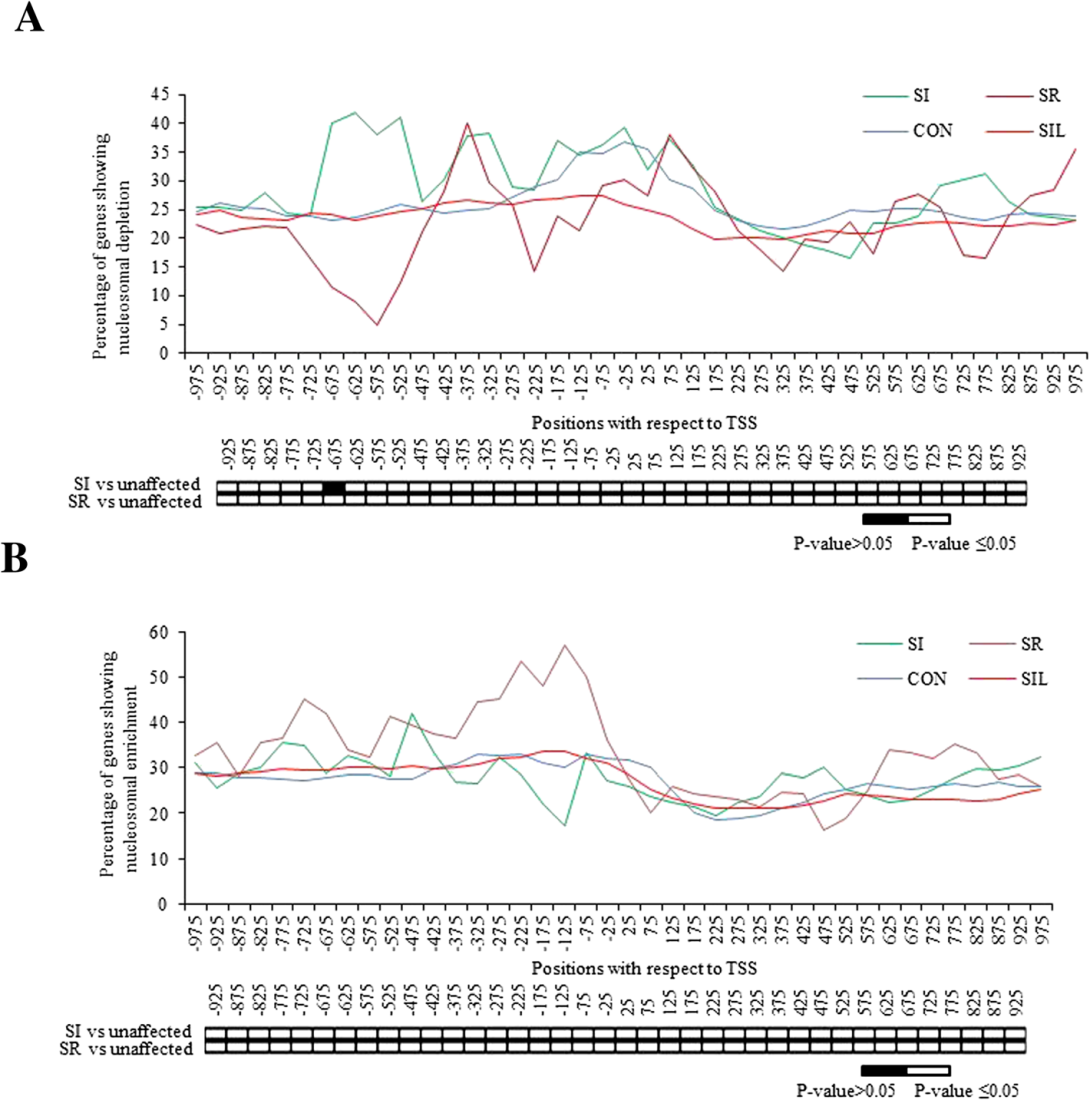
***Salicylic acid (SA) induced nucleosomal remodeling is more frequent in the SA responsive genes.*** The figure represents percentage of genes showing remodeled nucleosome out of total nucleosomes under any of the two conditions (**A** shows the nucleosomal depletion and **B** shows the nucleosomal enrichment respectively upon SA treatment) in −1000 bp to +1000 bp long regions in all the four groups. X-axis represents the positions with respect to Transcription start site (TSS) where −75 means −51 to −100. Y-axis represents percentage of genes having remodeled nucleosome at the particular position. Below the graph is the statistical significance data for each position when compared for different groups as mentioned beside the map (Unaffected group constitutes ‘CON’ and ‘SIL’ groups together) at P-value ≤0.05.

To gain better insights about the correlation of nucleosomal remodeling in promoters with the SA-mediated response, specific change in nucleosomal remodeling (i.e. either depletion or enrichment) at the TSS was ascertained in all these groups on SA treatment. For this, the total numbers of genes, showing nucleosomal occupancy under any condition, were retrieved and the percentage of the genes showing either nucleosomal depletion or enrichment on SA treatment around −1000 to +1000 bp region with respect to TSS was calculated. Mapping of the cumulative data showed interesting patterns. It was found that the region downstream of TSS show comparatively less remodeling in all the four groups (Figure 4). The CON and SIL groups show less nucleosomal remodeling even at the upstream region (−1000 bp with respect to TSS), where the percentage of genes undergoing nucleosomal depletion or enrichment increases only marginally near TSS (Figure 4). The SI and SR groups show a significant and mutually opposite pattern. The SI and SR groups show nucleosomal depletion (Figure 4A) and nucleosomal enrichment (Figure 4B), respectively, at the complete promoter region as compared to CON and SIL groups, which are statistically significant at P-value ≤0.05. Interestingly, the SI group also shows minimum nucleosomal enrichment at TSS (Figure 4B), and the SR group shows minimum nucleosomal depletion at TSS (Figure 4A) on SA treatment. Thus, the analysis establishes a correlation between the nucleosomal remodeling at proximal promoters and their expression on SA treatment.

To understand the reason behind distinct nucleosomal remodeling profiles of different groups, collective expression profile of these groups was obtained by annotating these groups on the basis of their functions. It is found that most of the CON genes were significantly expressed in different metabolic pathways, while most of the SIL genes were significantly expressed in reproductive and floral development pathways (see Additional file 10). This indicates that the SIL genes, in general, may be involved in some specific pathways, thus allowing minimum disturbance at their promoter by this treatment. Similarly, the SI genes show significant involvement in SAR pathway, SA mediated signaling pathways, ROS pathway, response to hypoxia and other relevant pathways while SR genes showed involvement in amino acid biosynthesis and metabolic processes, photosynthesis etc.

### Nucleosome remodels at the promoter locus of *PR1*

The data analysis at the minimal promoter region of the *PR1* gene shows the presence of a nucleosome upstream TSS, on TATA-box which is removed under SA-treated conditions (Figure 1A). The absence of the *PR1* core promoter nucleosome is depicted due to the MNase1 digestion strategy which preferentially digests the open chromatin in regions such as promoters of active genes [15,50,52]. Transcriptional activation requires the assembly of pre-initiation complex (PIC) upstream TSS, and the nucleosomal removal is sometimes required for active transcription [53]. In the past, studies have shown that the nucleosome is lost in the promoter region of *Pho5* (encoding a repressible acid phosphatase in yeast) gene during transcription activation in yeast [15,52,54,55] and in humans [50,51] where it is hyperacetylated before being lost. Similarly, Interferon β-promoter is activated on nucleosome sliding by viral infection [56]. Li *et al.* have also shown the same for the beta-phaseolin gene [36]. Till date, the nucleosomal loss has been shown to accompany gene activation by either sliding of nucleosomes or eviction of nucleosomes.

**Figure 5:**
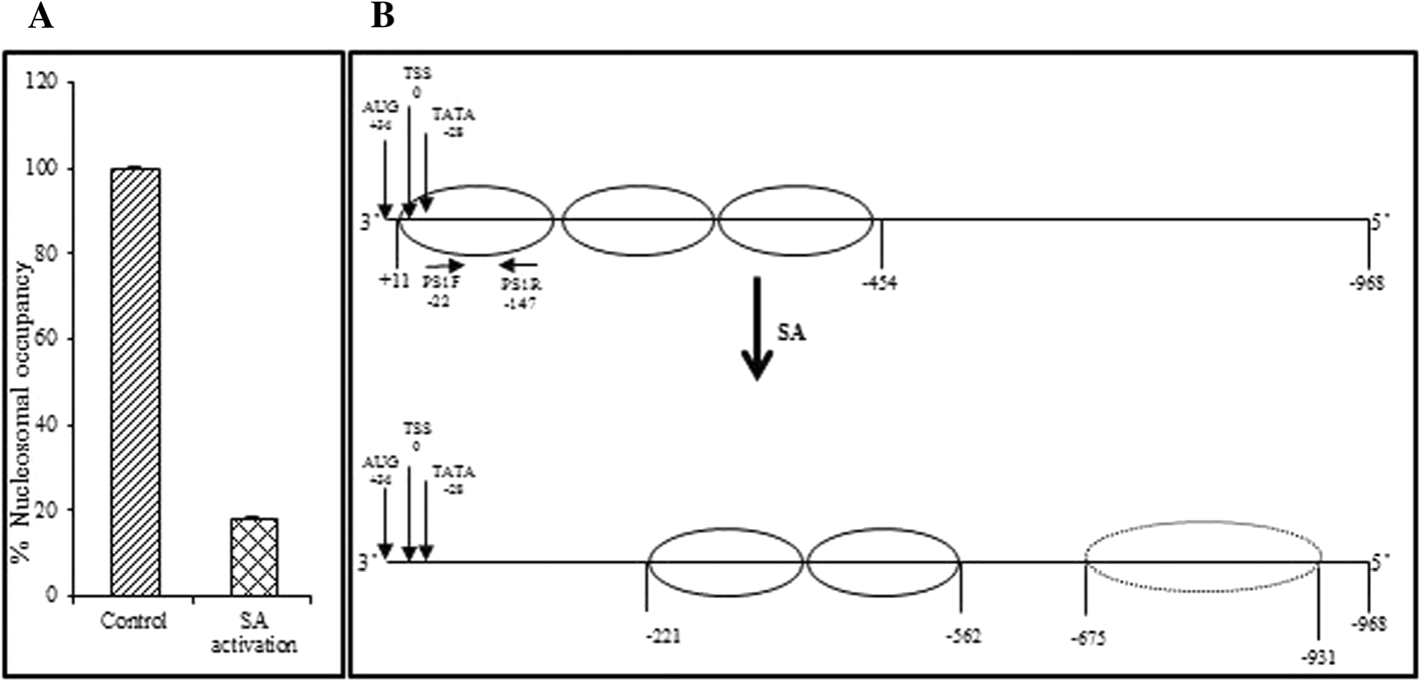
***Nucleosome remodeling at the At2g14610 locus prior and post Salicylic acid induction.*** **A**– Validation of the *PR1* promoter nucleosome remodeling around At2g14610 promoter by QPCR. The bar with diagonal stripes represents percentage of nucleosomal occupancy around TATA-box assigned as 100% and the checkered bar represents the percentage nucleosomal occupancy around TATA-box upon SA activation. **B**– Schematic representation of nucleosomal remodeling around At2g14610 promoter regulatory region as per Figure 1A and 5A. PS1F (−22) and PS1R (−147) are the primer pairs used for the QPCR validation.

This data analysis was validated by QPCR analysis using primers designed around the core promoter region at the *PR1* locus using a mononucleosomal template (see Additional file 3, primer sequences cover a −22 to −147 bp region with respect to TSS). The analysis shows a 5- to 6-fold reduction in amplification in case of the SA-treated conditions as compared to the control sample. This indicates a loss of nucleosomal signal from the *PR1* promoter locus (Figure 5A). Thus indeed, the QPCR results agree with the model deduced using tiling microarray at the stringency level of ≥−1.0 (Figure 5B).

The nucleosomal repositioning in the 5′ regulatory region is found not only in *PR1*; rather, it is reproducible in other genes of the SI group. The -1000 bp to 1000 bp region of all the SI genes was mapped for the remodeling of nucleosomes (both depleted and enriched) over the entire region on SA treatment. The data (see Additional file 1) show that while the TSS remains largely depleted of nucleosomes even under uninduced conditions (represented as black colour), about two-third of the remaining nucleosomes undergo remodeling on SA treatment (represented as green colour for depletion and red colour for enrichment), with only a few genes showing no change in the presence of the nucleosome around TSS (represented as cyan colour). Thus, the results indicate that the nucleosome remodeling does not limit only to the *PR1* gene but is applicable to the complete SI group in *A. thaliana*.

### NPR1 plays distinct role in the nucleosomal remodeling around the core promoter region of the SA-regulated genes

**Figure 6:**
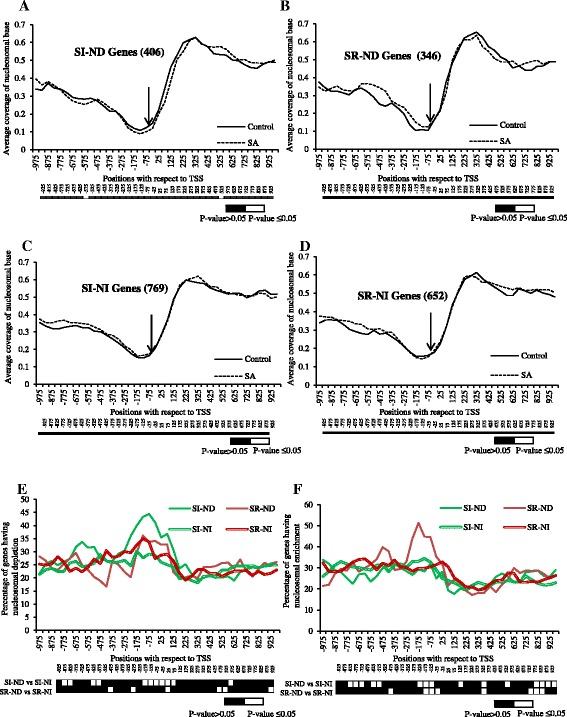
***NPR1 dependent genes function by nucleosome remodeling.*** Coverage of −1000 bp to +1000 bp long region by nucleosome was done for SA induced NPR1 dependent – SI-ND **(A)**, SA repressed NPR1 dependent – SR-ND **(B)**, SA induced NPR1 independent – SI-NI **(C)** and SA repressed NPR1 independent – SR-ND genes **(D)**. The numbers of genes taken in each group is given in parentheses. The −25 position is shown with arrow. Below each figure is shown the comparative statistical significance difference for each window. **E)** and **F)** represent percentage of genes showing nucleosomal depletion and enrichment, respectively, out of total nucleosomes upon SA treatment in −1000 bp to +1000 bp long regions in all the four groups **(A-D)**. X-axis represents the positions with respect to TSS where −75 means −51 to −100 and Y-axis represents either average coverage of nucleosomal base **(A-D)** or percentage of genes getting remodeled at each position **(E-F)**. Below the graph is the statistical significance data for each position when compared for different groups as mentioned beside the map (Unaffected group constitutes ‘CON’ and ‘SIL’ groups together) at P-value <0.05.

The SAR pathway is regulated by chromatin remodeling [57] and works in both an NPR1-dependent (ND) and NPR1-independent (NI) manner downstream to SA induction. Since *PR1* is an ND gene, it was interesting to see whether the nucleosome depletion at the −25 window (i.e. 0 to -50 bp with respect to TSS) in the SI genes is affected by the presence of NPR1. Thus, SA-regulated -ND and -NI genes were identified (GSE 51626, see Additional file 9) [39]. Further, it was observed that ~80% of the SA-regulated genes used to study nucleosomal remodeling patterns (Figure 3C and D) are also present in the list of SA-regulated genes selected by two-way ANOVA (at P-value ≤0.05). A list showing the functional GO groups to which each gene significantly contribute was generated, when these genes were subjected to annotation followed by singular enrichment analysis under agriGO software (see Additional file 11). The analysis confirms the expression profile of each subset of the genes to their respective group; that is, while both groups are involved in the defense pathways; ND genes show significantly more involvement in defense pathways than the NI genes (see Additional file 11). Each group is further divided into two: SI and SR. Interestingly, the examination of nucleosomal coverage in these subgroups in the −1000 to +1000 bp regions with respect to TSS in a window of 50 bp shows that the average nucleosomal coverage at −25 window does not change significantly under SA-treated condition as compared with the control condition in all four groups (i.e. SI-ND, SR-ND, SI-NI, and SR-NI) (Figure 6A-D). So, the graphs showing percentage of genes undergoing either nucleosomal depletion (Figure 6E) or nucleosomal enrichment (Figure 6F) on SA treatment were further plotted. Statistically significant nucleosomal depletion just above the TSS (−25 window) in SI-ND genes as compared with the SI-NI genes was observed, which indeed, extends from 75 to −175 windows at P-value ≤0.05 (Figure 6E). Interestingly, significant nucleosome depletion in SR-ND genes around TSS as compared to SI-NI genes (−75 to 25 windows) was also observed at P-value ≤0.05; however, not in comparison to SR-NI genes. Similarly, statistically significant nucleosomal enrichment above the TSS (−75 to −175 windows) in SR-ND genes as compared to the SR-NI genes was observed at P-value ≤0.05. No nucleosomal enrichment was observed in SI-ND genes; rather, a significant decline in nucleosome enrichment in SI-ND genes (−125 to −225 windows) was observed as compared to SI-NI genes at P-value ≤0.05. Thus, the results show that both the nucleosomal depletion in SI-ND genes and the nucleosomal enrichment in SR-ND genes in response to SA induction correlate to the presence of NPR1. Overall, it suggests a distinct role of NPR1 in chromatin remodeling in SA-responsive genes in *A. thaliana*. However, the nucleosome depletion observed in SR-ND genes over SI-NI genes does not seem to correlate to the presence of NPR1.

**Figure 7:**
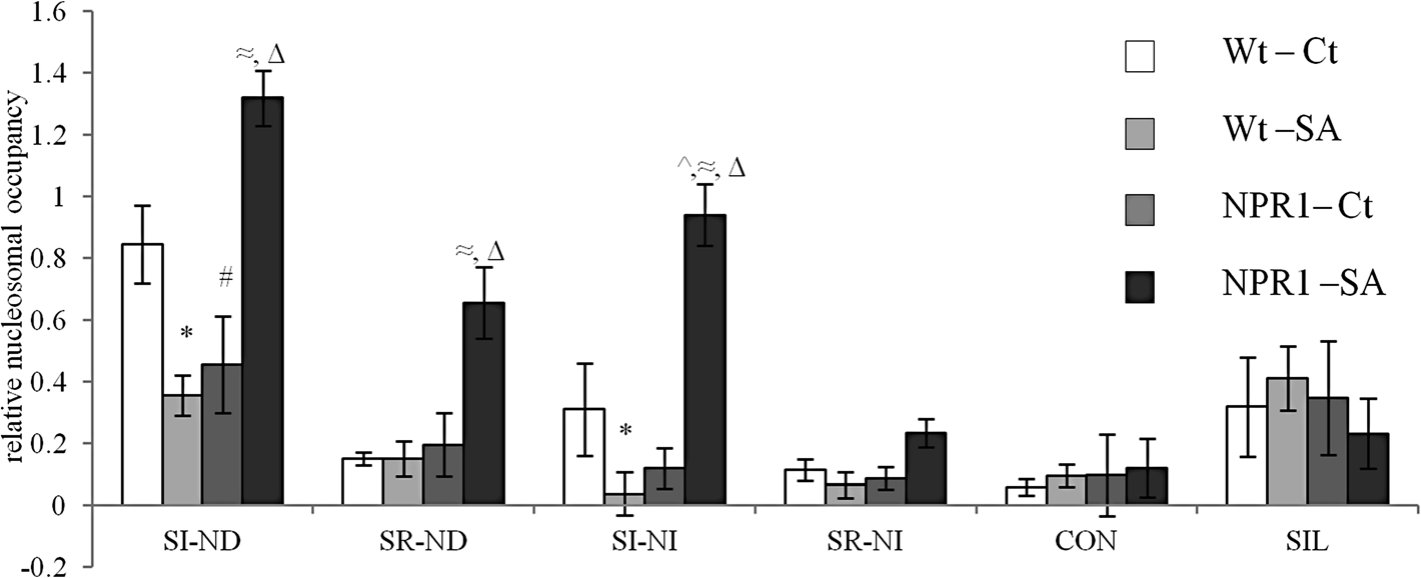
***NPR1 plays distinct role in the nucleosomal remodeling around the core promoter region of the SA regulated genes.*** Figure shows average relative nucleosomal occupancy in Col-0 (c) and *npr1-1* mutants (n) under untreated (w) and treated (s) conditions of three genes from each group after normalization with a set of five internal control genes and with SE. ‘*, #, ^, ≈ and Δ’ denote the significance of the change in nucleosomal occupancy values among Wt-Ct–Wt-SA, Wt-Ct–NPR1-Ct, Wt-Ct–NPR1-SA, Wt-SA–NPR1-SA and NPR1-Ct–NPR1-SA pairs respectively, as per Student’s *t*-test at p ≤0.05. No significant changes were found in the values among Wt-SA–NPR1-Ct pair in all groups. Complete Student’s *t*-test table has been given for all pair-wise comparisons among Wt-Ct, Wt-SA, NPR1-Ct and NPR1-SA for each gene class as Additional file 12.

The result based on the role of NPR1 in nucleosomal remodeling around the core promoter region has been further cross-validated. The nucleosomal occupancy at the core promoter and corresponding relative transcript abundance of three representative genes each from all the six categories under control and SA-treated conditions in two-week old seedlings of both wild-type (Col-0) and *npr1-1* mutant (Figure 7 and Additional file 1) has been checked. Based on the normalization with the internal control genes set, each group shows average expression profile according to its class. Our results show that the remodeling of the nucleosome at the promoter region in these different groups has characteristic differences (Figure 7 and Additional file 12). First, the CON, SIL, and SR-NI groups do not show any statistically significant change in nucleosomal occupancy at the minimal promoter in both wild-type and *npr1-1* mutant under either control or SA-treated condition at P-value ≤ 0.05 (Figure 7). SI-ND and SI-NI genes show significant remodeling of the nucleosome at minimal promoter post SA treatment either in the presence or in the absence of NPR1 at P-value ≤ 0.05; however, SR-ND genes show significant remodeling of the nucleosome at minimal promoter post SA treatment only in the absence of NPR1. Both SR-ND and SR-NI classes do not show any differences in the nucleosome occupancy at minimal promoter in Col-0 post SA treatment. Interestingly, in all the four SA-regulated classes (SI-ND, SR-ND, SI-NI, and SR-NI), treatment of SA causes a statistically significant increase in nucleosomal occupancy over the minimal promoter loci in the absence of NPR1 at P-value ≤0.05. NPR1 has long been known to mediate the plant defense pathways through SA and JA cross-talk [58]. Further, it is well understood that NPR1 accumulates in the nucleus post SA treatment [59] and associates with TGA transcription factors to regulate SA-mediated transcription [60,61]. An increase in the nucleosome occupancy in SA-regulated genes in the absence of NPR1 (Figure 7) indicates that NPR1 has a general role in chromatin remodeling in all SA-regulated genes post SA treatment. However, exact role of NPR1 in SA-mediated response is not clear from the present study and needs further investigation to augment our current understanding about the role of NPR1 in the global nucleosomal remodeling in response to SA.

### ABRE elements and MYB elements are enriched in NORs of SI genes, while W-box and TGACG/C motif are enriched in NFRs

**Figure 8:**
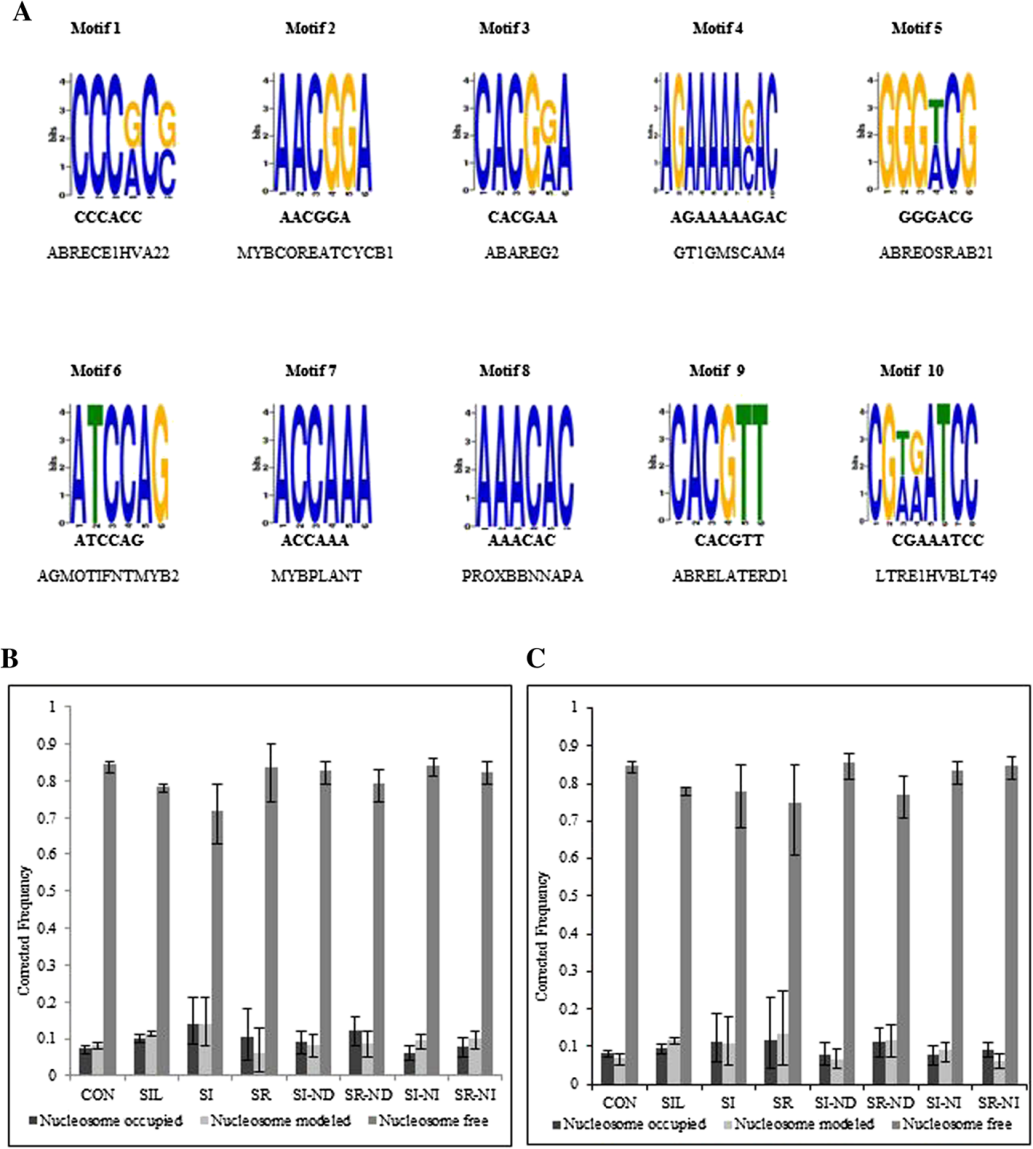
***Motif analysis in the promoter region of the Salicylic Acid Induced group.*** **A** – The top 10 motifs generated using MEME software. **B** and **C**– The W Box and TGA box occupancies in different groups. X-axis shows different groups of genes analyzed. The error bar represents 95% Binomial Confidence Interval and Y-axis shows the relative corrected frequency of respective motifs in each group with respect to the corrected frequency for complete promoter regions.

Next, some *cis*-regulatory motifs were identified in the remodeled nucleosomal region in the promoters (1 kb upstream of TSS) of 111 SI genes (See Methods) and annotated as per PLACE database. These motifs include, at the top, motif 4, similar to GT1GMSCAM4, which interacts with GT-1 transcription factor to activate the pathogen- and salt-inducible gene SCaM-4 in *Glycine max* [62]. Motifs 1, 3, 5, 8 and 9 are similar to different ABRE motifs (Figure 8A) like ABRECE1HVA22, ABAREG2 element, ABREOSRAB21, PROXBBNNAPA and ABRELATERD1 (involved in ABA specific gene expression in barley [63] during plant embryogenesis and seed germination [64,65], as consensus sequence in wheat, rice and *Brassica napa* genes [66-68], and in regulating etiolation-induced activity in *A. thaliana* [69]) respectively. Motif 2, 6 and 7 are similar to MYB family motifs like MYBCOREATCYCB1, AGMOTIFNTMYB2 and MYBPLANT (involved in cell-cycle phase independent cyclin B1 gene activation [70], in the activation of a wound responsive gene [71], as a consensus sequence in phenylpropanoid biosynthesis pathway gene promoters [72]) respectively (Figure 8A). Motif 10 is similar to LTREHVBLT, a low temperature response element in the barley blt4.9 promoter [73]. The preferred presence of these motifs cannot be attributed to the biasness caused by the use of MNase1, as then it would not be found specifically in the SI genes.

Promoters of the genes co-regulated with *PR1* in the SAR pathway have been reported to be highly enriched in W-box motif TTGAC [31], providing binding sites to WRKY transcription factors. Another pentameric motif TGACG/C, overlapping core W motif TGAC, is not very common in *PR1* regulon genes. However, both TGA factors and WRKY transcription factors are reported to regulate SA response by binding TGACG motif and W-box, respectively, in the *PR1* promoter [61,74] and globally [75,76]. Therefore, it was interesting to identify the actual presence of both these motifs in context of nucleosomes. Surprisingly, none of these motifs was observed as enriched in the remodeled region by MEME (Figure 8A). So, the presence of the W-box motif and TGAGC/C motif was examined in the promoter region of all groups, namely, CON, SIL, SI, SR, SI-ND, SR-ND, SI-NI, and SR-NI. Both of the motifs were identified as enriched in the NFRs in the promoters of all the groups (Figure 8B-C and Additional file 13) invariably. Only a very small fraction of W-box or TGA motifs was present in either the nucleosomal or remodeled region. Interestingly, enrichment of the TGA motif was considerably higher in both ND and NI groups as compared to other groups (Figure 8C). Both motifs were significantly depleted in the SIL group as compared with other groups (Figure 8B-C), reflecting the functional significance of these motifs that are enriched in the active group. Thus, it is concluded that both the W-box and TGA motifs are significantly enriched in NFR. TGA factors are involved in regulating basal expression of SAR genes by either repressing the locus or promoting its basal expression [77,78], and they physically interact with as 1-like elements for *PR-1a* gene regulation [79]. Hence, the presence of W-box and TGACG motifs in NFR is interesting and needs to be validated regarding the affinity of WRKY transcription factors and TGA factors to bind their cognate sequence in nucleosomal and non-nucleosomal templates, respectively.

### Upstream activator region in the SR genes harbours many potential motifs involved in the plant defense pathway

The SR group shows characteristic nucleosomal remodeling by enrichment on SA treatment at an upstream region, that is, −600 to –800 bp upstream of TSS (Figure 3D). Using Lift and Motif enrichment methods, 40 common motifs were found to be enriched in SR over the SI group in the −600 to –800 bp promoter region, with enrichment fold change ≥2 (Table 1). On annotation of these motifs using PLACE database, it was found that many of these are involved in the plant defense pathway. Among such important motifs include three motifs similar to LS5ATPR1, one motif each similar to LS7ATPR1, AS1LIKECSHPRA, TGA1NTPR1A and PR2GCNT and two motifs similar to WBOXNTCHN48 (Table 1). LS5 and LS7 have been reported to provide binding sites to TGA2 and TGA1 respectively in *A. thaliana* [61,80]. TGA1NTPR1A and AS1LIKECSHPRA are reported as as1-like elements in tobacco and cucumber [81,82] respectively. Similarly, PR2GCNT and WBOXNTCHN48 are reported to be present in stress- and developmentally-regulated pathogenesis-related acidic tobacco β-1,3-glucanase gene [83] and an ethylene inducible tobacco class1 chitinase48 gene [84] respectively. Three motifs each similar to AGCBOXNPGLB and QARBNEXTA were also found, which were earlier reported in tobacco β-1,3-glucanase gene enhancer element similar to GCC element required for ethylene regulated transcription of *extA* gene in *B. napa* in response to wounding and tensile stress [85,86]. Many motifs similar to ABRE elements were found like ABREDISTBBNNAPA, ABREMOTIFIIIOSRAB16B, ABREMOTIFIOSRAB16B, ABRECE1HVA22, ABRE3HVA22 and ABRETAEM earlier identified in *B. napa*, rice, barley and wheat in relation to abscissic acid regulated transcription [64,65,68,87,88]. Likewise, several motifs similar to MYB2 binding elements were found — AGMOTIFNTMYB2, MYB26PS and MYBPZM (Table 1) which were linked to many defense associated pathways like phenylpropanoid pathway, flavonoid biosynthetic pathway, wound inducible pathway etc. [71,89,90]. Two motifs similar to the SORLIP2AT element were also found in our analysis [91]. Motifs similar to GARE2OSREP1, GAREHVAMY1, NDEGMSAUR, AUXREPSIAA4, D4GMAUX28, DRECRTCOREAT and DRE1COREZMRAB1 involved in other stress response pathways like auxin response pathway, gibberellic acid pathway and dehydration response pathway [92-101] were also identified. The cross-talk between SA, JA, auxin, gibberellin, and ABA has also been reported earlier to fine tune the plant stress responses [29,102-106]. Our study also indicates that SA induction is responsible for chromatin remodeling over the *cis*-regulatory elements that are involved in both the SA-mediated response of SA-induced genes and the JA- and ABA-mediated response of SA-repressed genes.

**Table 1:** **Overrepresented motifs in Salicylic acid repressed (SR) group in the -600 bp to -800 bp region upstream TSS**

**Consensus motif**	**OE**	**LIFT**	**PLACE ID**
GCCATG	33	6.404850381	NDEGMSAUR
CGATCC	27.5	5.337375318	AGMOTIFNTMYB2
CACTCG	27.5	5.337375318	ABREDISTBBNNAPA
ACCCGT	22	4.269900254	QARBNEXTA
CACGCT	22	4.269900254	ABREMOTIFIIIOSRAB16B
GTCGAC	18	6.404850381	DRECRTCOREAT
CGATGG	15	5.337375318	ABRECE1HVA22
TGTACG	13.2	6.404850381	ABRE3HVA22
AAGGTA	12.1	11.7422257	GARE2OSREP1
ACGGGT	12	4.269900254	QARBNEXTA
CGTACC	12	4.269900254	ABRE3HVA22
CGTGAC	12	4.269900254	LS5ATPR1
GCACTG	12	4.269900254	D4GMAUX28
ACTGAC	11	5.337375318	WBOXNTCHN48
CGTAGC	10.5	7.472325445	LS5ATPR1
GTCACG	10.5	7.472325445	LS5ATPR1
TAGCCG	10.5	3.736162722	AGCBOXNPGLB
TATACG	9.9	9.607275572	RSRBNEXTA
TGGACC	9	6.404850381	UPRE1AT
TAGGTC	8.4	7.472325445	MYB26PS
AGGCCC	8	8.539800509	SORLIP2AT
CTAGAC	7.7	3.736162722	SURECOREATSULTR11
CACACG	7.5	5.337375318	QARBNEXTA
CGGCTT	7.5	5.337375318	PR2GCNT
GCAGCA	7.5	5.337375318	D4GMAUX28
TGTGCC	7.5	5.337375318	RNFG2OS
ATGGCC	7.5	5.337375318	CAATBOX2
GTTCAG	7.2	6.404850381	WBOXNTCHN48
GCATAG	7.2	6.404850381	LS7ATPR1
TATCGG	7.2	6.404850381	GAREHVAMY1
AATGCG	6.6	3.202425191	AS1LIKECSHPRA
GTATGC	6.6	3.202425191	SORLIP4AT
CCCATC	6	4.269900254	AUXREPSIAA4
CTCTCG	6	4.269900254	DRE1COREZMRAB17
AACCGC	6	4.269900254	AGCBOXNPGLB
ACAGGC	6	4.269900254	ANAERO5CONSENSUS
AGACGC	6	4.269900254	AGCBOXNPGLB
AGGGCA	6	4.269900254	ANAERO5CONSENSUS
CGTTGG	6	4.269900254	NDEGMSAUR
GGACCA	6	4.269900254	ABREMOTIFIOSRAB16B
GGTACT	6	5.337375318	ABREMOTIFIOSRAB16B
GTGTCC	6	4.269900254	ABRETAEM
GTTAGG	6	4.269900254	MYB26PS
TCTCGA	6	5.337375318	TGA1ANTPR1A

## Conclusions

Perturbations change the transcriptional profile of an organism. These changes require both silencing and activation of certain genes and, thus, demand chromatin remodeling of such gene loci. In our study, conspicuous nucleosomal remodeling is found in the promoter region of salicylic acid regulated genes, where, under any of the two conditions, 40% of the chromatin is occupied by nucleosomes. The nucleosomal coverage is higher in 3′ coding regions as compared to the non-coding regions. Likewise, the nucleosome coverage is higher over the entire chromosome except the centromeric region with marginally higher coverage in the pericentromeric regions. The nucleosomal remodeling at the promoter region in constitutive and silent groups remains insensitive to the salicylic acid treatment, whereas both salicylic acid induced and salicylic acid repressed groups show nucleosomal depletion and enrichment, respectively, on salicylic acid treatment. The nucleosomal depletion in the promoter region of the salicylic acid induced genes is a general feature of the group. A distinct role of Non-expresser of PR1 (NPR1) in salicylic acid regulated remodeling is observed. The salicylic acid induced-NPR1dependent genes operate through nucleosomal depletion, and salicylic acid repressed-NPR1 dependent genes operate through nucleosomal enrichment in response to salicylic acid induction in an NPR1-dependent manner. All the four groups, that is, salicylic acid induced-NPR1 dependent, salicylic acid repressed-NPR1 dependent, salicylic acid induced-NPR1 independent, and salicylic acid repressed-NPR1 independent, show significant nucleosomal enrichment in the absence of NPR1 on salicylic acid treatment.

The remodeled nucleosomal regions in salicylic acid induced groups harbour many important motifs that are known to be enriched in the genes involved in defense mechanisms such as ABRE elements, MYB box etc. Nevertheless, prominent motifs in the salicylic acid pathway, that is, W-box and TGACG/C, are enriched in nucleosome free regions of the promoter region. Even the upstream region of salicylic acid repressed groups harbours many motifs that are involved in defense pathways.

Overall, this study reports, for the first time, the genome-wide effects of salicylic acid treatment on the chromatin architecture of *A. thaliana.* It also interprets the enrichment of relevant motifs with respect to overall nucleosomal positioning in the promoter regions of salicylic acid regulated genes.

### Availability of supporting data

The datasets supporting the results of this article are available in the NCBI GEO repository as, http://www.ncbi.nlm.nih.gov/geo/query/acc.cgi?acc=GSE25553 and http://www.ncbi.nlm.nih.gov/geo/query/acc.cgi?acc=GSE51626.

## Additional files

Additional file 1:
**Additional Information.pdf containing description of additional figures as follows:** **Figure S1.** Mononucleosomes preparation from 14 days old untreated or Salicylic acid treated *A. thaliana* seedlings. **Figure S2.** Box plots of log2 transformed values for control and Salicylic acid treated replicate arrays used for the classification of four different groups. **Figure S3.** Schematic representation of nucleosomes either present or absent in both the cases and their validation of nucleosome occupancy in *A. thaliana* genome using QPCR analysis. **Figure S4.** Validation of nucleosomal remodeling by mapping of Salicylic acid induced genes. **Figure S5.** NPR1 plays a distinct role in the nucleosomal remodeling around the core promoter region of the Salicylic acid regulated genes. Additional file 2:
**Nucleosomal Coverage for all the five chromosomes in** ***A. thaliana.***
Additional file 3:
**Sequences of the probes and primers used in this study.**
Additional file 4:
**Regions showing presence and absence of nucleosomes used for QPCR verification of nucleosomal occupancy over four loci having a nucleosome under control conditions and four other loci lacking any nucleosome under control conditions as per Additional file** 1 **: Figure S3.**
Additional file 5:
**Number of distinct and fuzzy nucleosome at different stringent levels.**
Additional file 6:
**Nucleosome remodeling post Salicylic acid (SA) induction.**
Additional file 7:
**Percentage of Nucleosomal occupied Regions (NORs) in different region of total genes of** ***A. thaliana*** **Genomes.**
Additional file 8:
**Nucleosome coverage at the centromeric and pericentromeric region on all the chromosomes of** ***A. thaliana*** **.**
Additional file 9:
**List of Accession number of the genes under each category as per Figures** 3 **and** 6 **.**
Additional file 10:
**GO analysis of the four groups of genes as per Figure** 3 **.**
Additional file 11:
**GO analysis of NPR1-dependent and NPR1-independent group of genes as per Figure** 6 **.**
Additional file 12:
**Student** ***t*** **-test values for all the pairs for Figure** 7 **.**
Additional file 13:
**Calculation of the TTGAC and TGACG/C motif enrichment in promoters of different group of genes.**

## Abbreviations

SA: Salicylic acid
*PR-1a*: Pathogenesis related gene-1a (in tobacco)
PR1: Pathogenesis related gene 1 (in *A. thaliana*)
MNase1: Micrococcal nuclease1
TSS: Transcription start site
SAR: Systemic acquired resistance
NPR1: Non- expresser of PR1
CON: Constitutive
SIL: Silent
SI: SA induced
SR: SA repressed
ND: NPR1-dependent
NI: NPR1- independent
SI-ND: SA induced-NPR1 dependent
SR-ND: SA repressed-NPR1 dependent
SI-NI: SA induced-NPR1 independent
SR-NI: SA repressed-NPR1 independent
NOR: Nucleosome occupied region
NFR: Nucleosome free region
