# Supplementary material for: Global nucleosome positioning regulates salicylic acid mediated transcription in Arabidopsis thaliana
Source: BMC Plant Biol. 2015 Jan 21;15:13. doi: 10.1186/s12870-014-0404-2 (PMC4318435; doi:10.1186/s12870-014-0404-2)
Supplement: Additional file 1: — Additional Information.pdf containing description of additional figures as follows: Figure S1. Mononucleosomes preparation from 14 days old untreated or Salicylic acid treated A. thaliana seedlings. Figure S2. Box plots of log2 transformed values for control and Salicylic acid treated replicate arrays used for the classification of four different groups. Figure S3. Schematic representation of nucleosomes either present or absent in both the cases and their validation of nucleosome occupancy in A. thaliana genome using QPCR analysis. Figure S4. Validation of nucleosomal remodeling by mapping of Salicylic acid induced genes. Figure S5. NPR1 plays a distinct role in the nucleosomal remodeling around the core promoter region of the Salicylic acid regulated genes. [file 12870_2014_404_MOESM1_ESM.pdf]

Additional figure 1: Mononucleosome preparation from 14 days old untreated or Salicylic acid (SA) treated *A. thaliana* seedlings

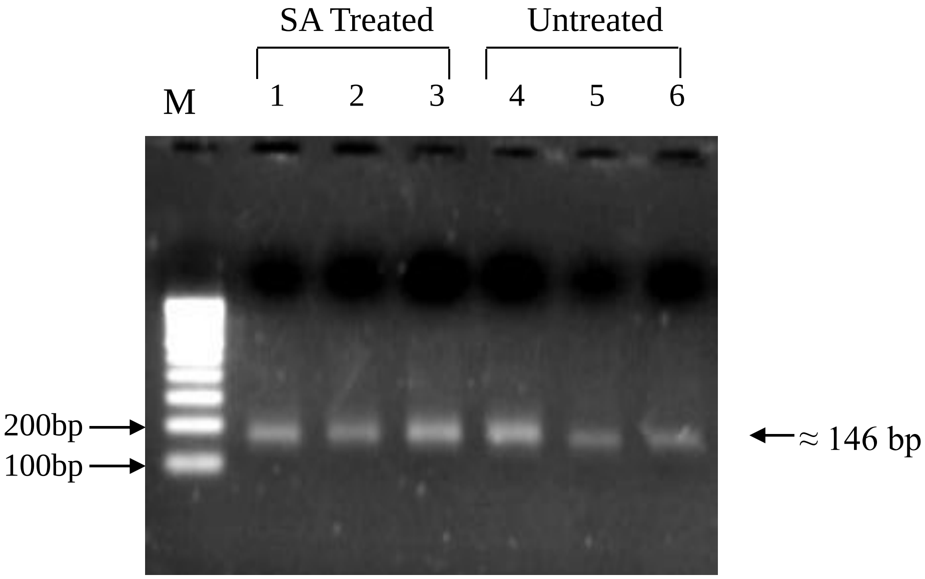

Additional figure 1: Label 1-3 represents the mononucleosome prepared from Salicylic acid treated samples and 4-6 represents the mononucleosome prepared from untreated samples used for tiling microarray experiments.

Additional figure 2: Box plots of log2 transformed values for control and Salicylic acid treated replicate arrays used for the classification of four different groups.

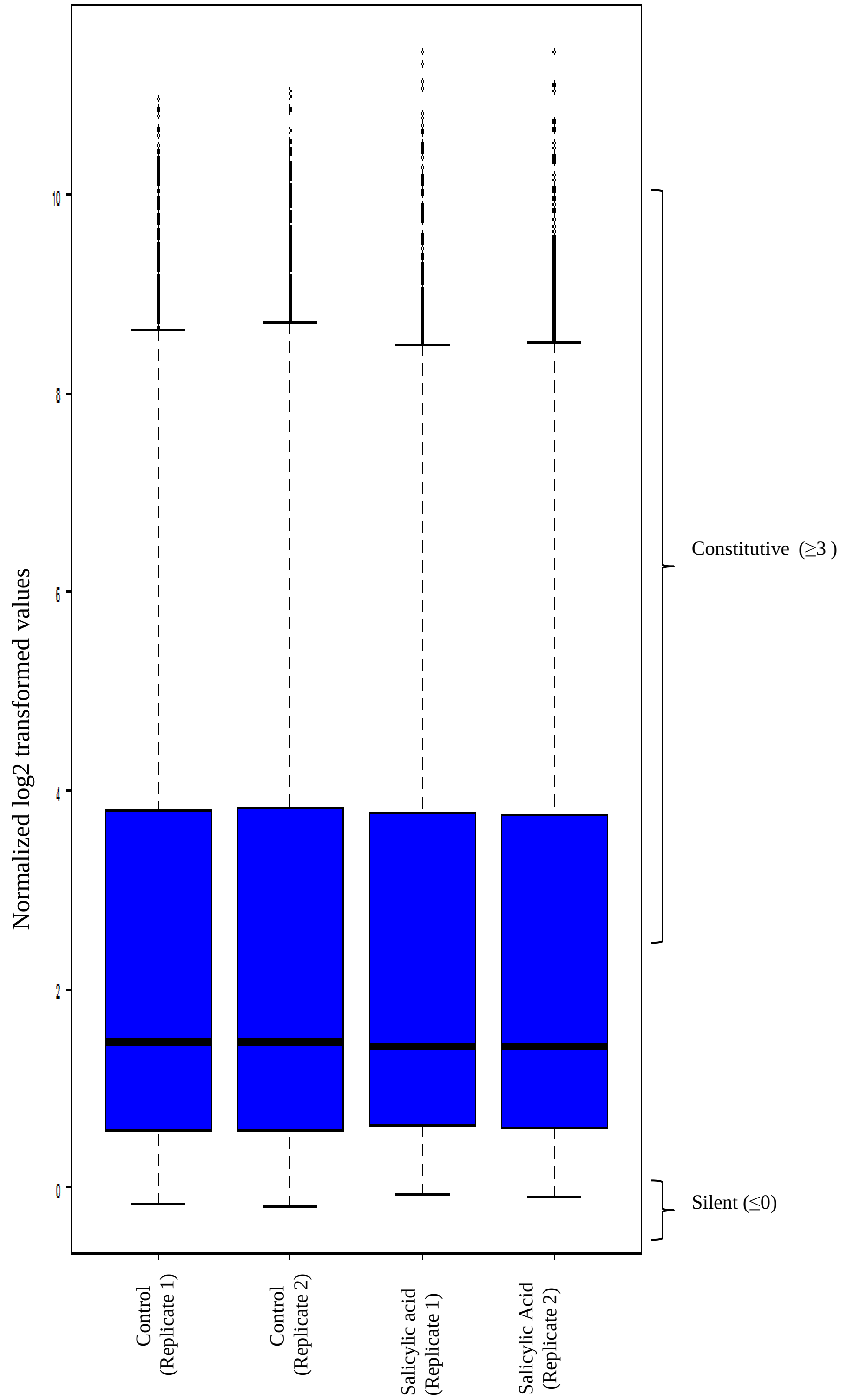

After Robust Multi-array Average performed under AffylmGUI R package , Box plot log-intensity distributions were plotted for array comparison where X axis indicates the control (Replicate 1, Replicate 2) and salicylic acid treatment (Replicate 1, Replicate 2) and Y axis indicates the normalized log2 transformed values of all the arrays.

Additional figure 3A: Nucleosome present at the locus under both untreated and Salicylic acid treated conditions

At1g06970 (Forward): ATCHX14

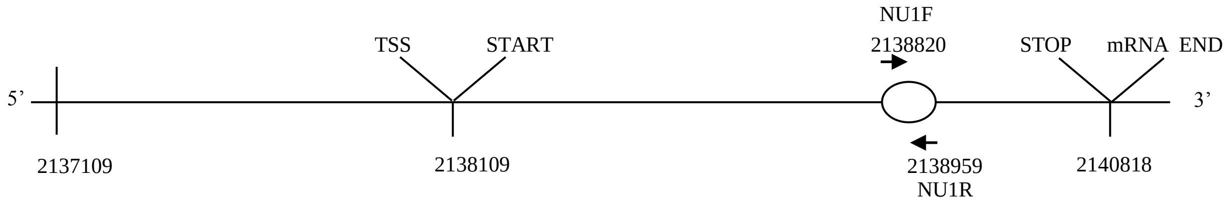

At2g39360 (Reverse): F12L6.2; Protein kinase superfamily protein

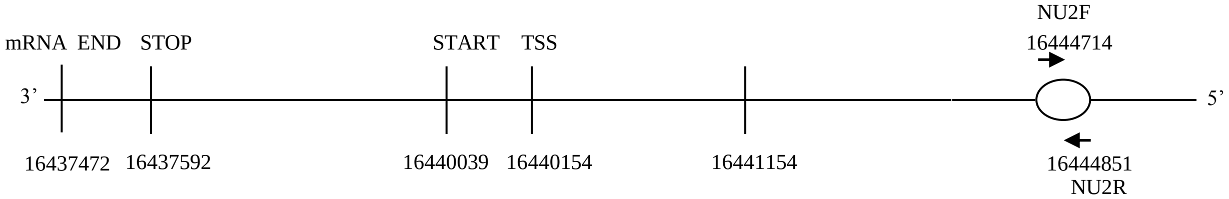

At4g01350 (Forward): F2N1.27; Cysteine/Histidine-rich C1 domain family protein

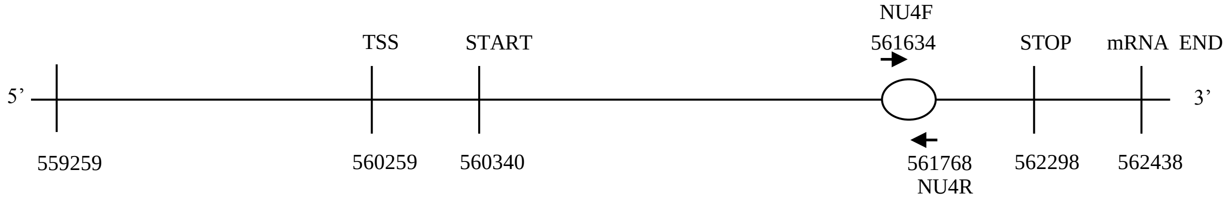

At5g59000 (Forward): K19M22.19; RING/FYVE/PHD zinc finger superfamily protein

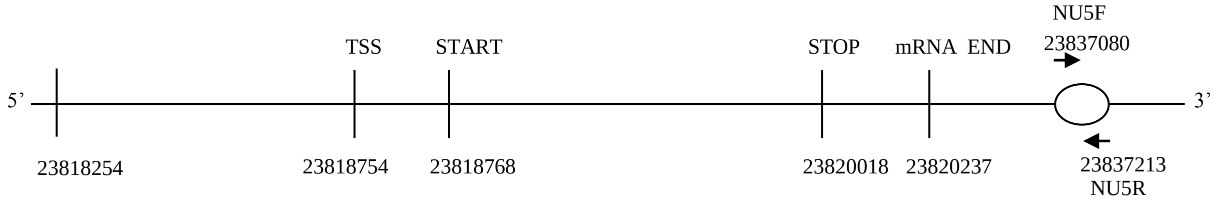

Additional figure 3A: The figure depicts the schematic diagram of all the four loci (present in exonic region) selected for the validation of the tiling microarray data for the presence of nucleosomes under control conditions. Each diagram shows its transcription start site (TSS), translation start site (START), translation termination site (STOP), mRNA transcription stop site (mRNA END), nucleosome on the strand (oval), forward and reverse primer positions (forward and reverse arrows along with the primer name as given in Additional table 1). Along with each point is mentioned its genomic position (in 7 digit numerals).

Additional figure 3B: Nucleosome absent at the locus under both untreated and Salicylic acid treated conditions

At1g53790 (Reverse): T1820.3; F-box and associated interaction domains-containing protein

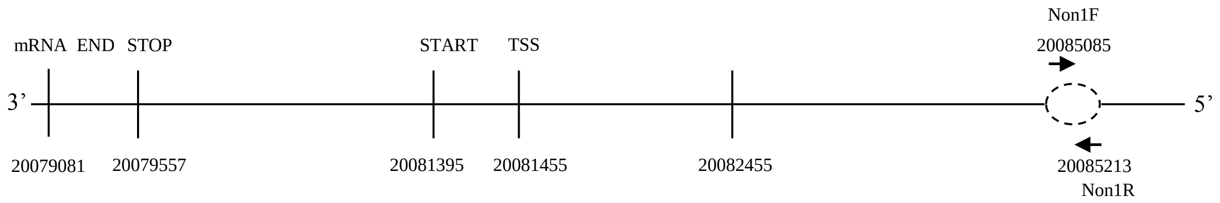

At2g28650 (Reverse): ATEXO70H8; A member of EXO70 gene family

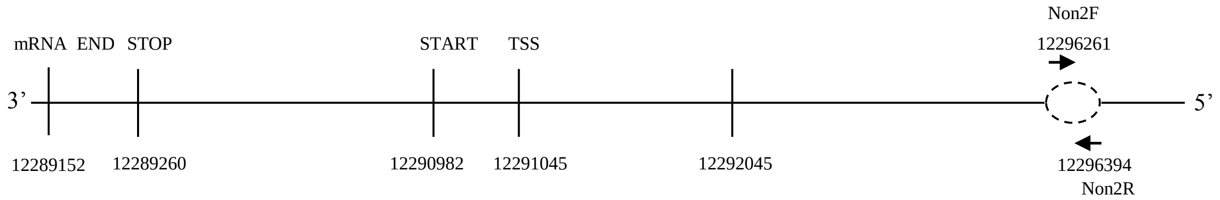

At3g55430 (Reverse): T22E16.90; O-Glycosyl hydrolases family 17 protein

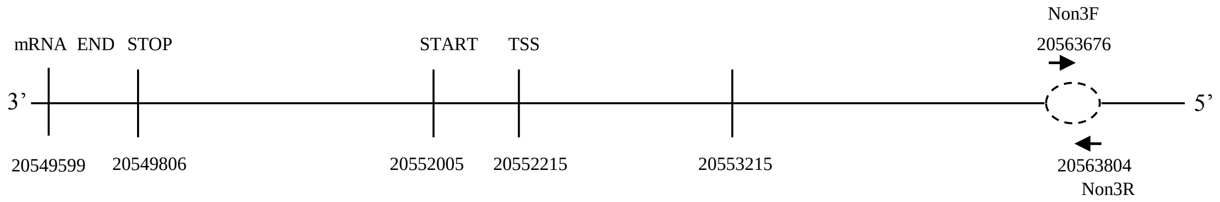

At4g07408 (Forward): Unknown protein

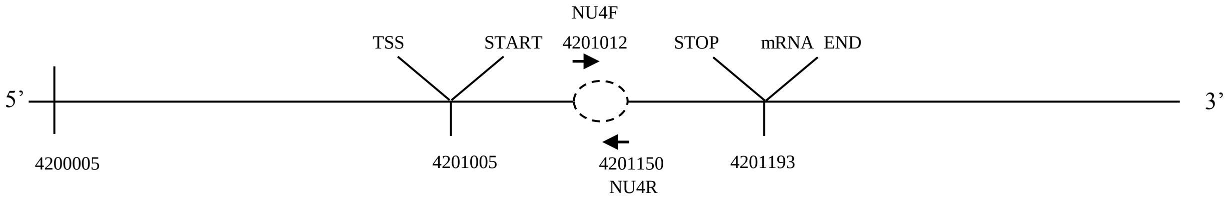

Additional figure 3B: The figure depicts the schematic diagram of all the four loci (present in intergenic-5'UTR region – At1g53790, exon-5'UTR region – At2g28650, completely intergenic – At3g55430 and completely exonic region – At4g07408) selected for the validation of the tiling microarray data for the absence of nucleosomes under control conditions. Each diagram shows its transcription start site (TSS), translation start site (START), translation termination site (STOP), mRNA transcription stop site (mRNA END), nucleosome on the strand (oval in dash), forward and reverse primer positions (forward and reverse arrows along with the primer name as given in Additional table 1). Along with each point is mentioned its genomic position (in 7 digit numerals).

Additional figure 3C: Validation of nucleosome occupancy in *A. thaliana* genome using QPCR analysis

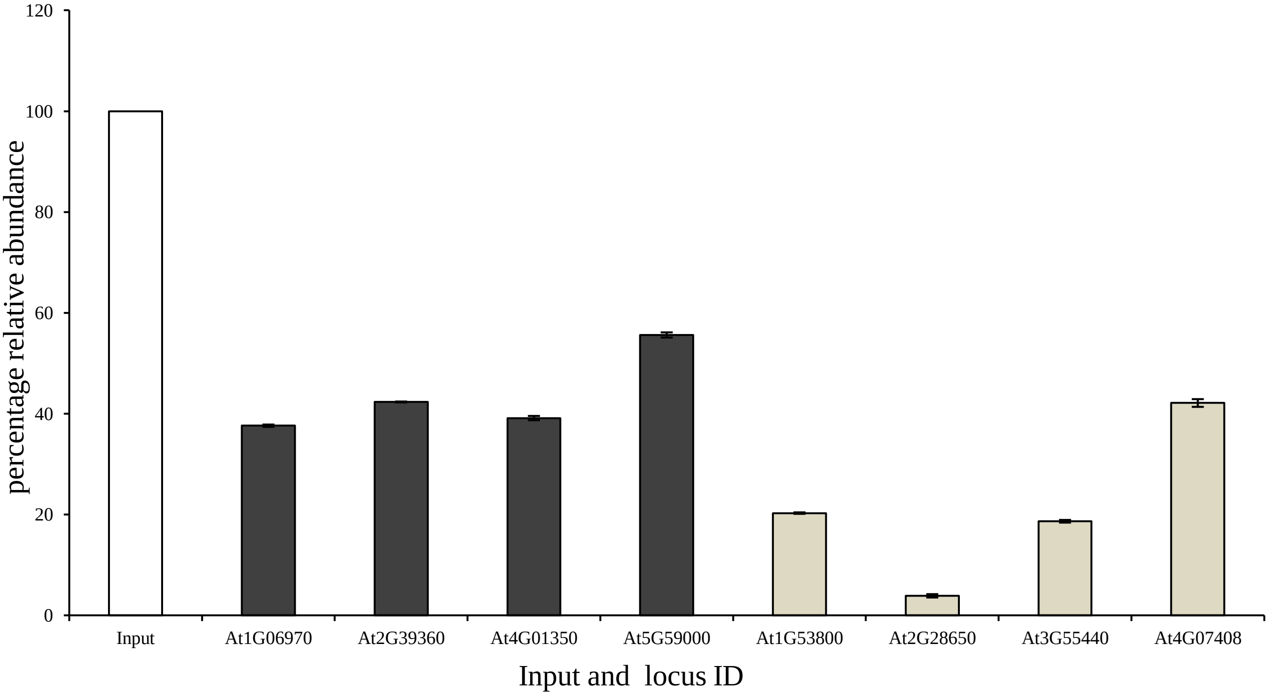

Additional figure 3C: QPCR analysis depicts the relative abundance of nucleosome with respect to genomic input both at the loci with nucleosomes (At06970, At2g39360, At4g01350, At5g59000, At1g53800 –black bars) and the loci without any nucleosome (At2g28650, At3g55440, At4g07408 –grey bars) under the tested conditions. Y-axis represents the percentage relative abundance.

Additional Figure 4: The nucleosomal repositioning in the 5’ regulatory region is a common feature of all SI genes.

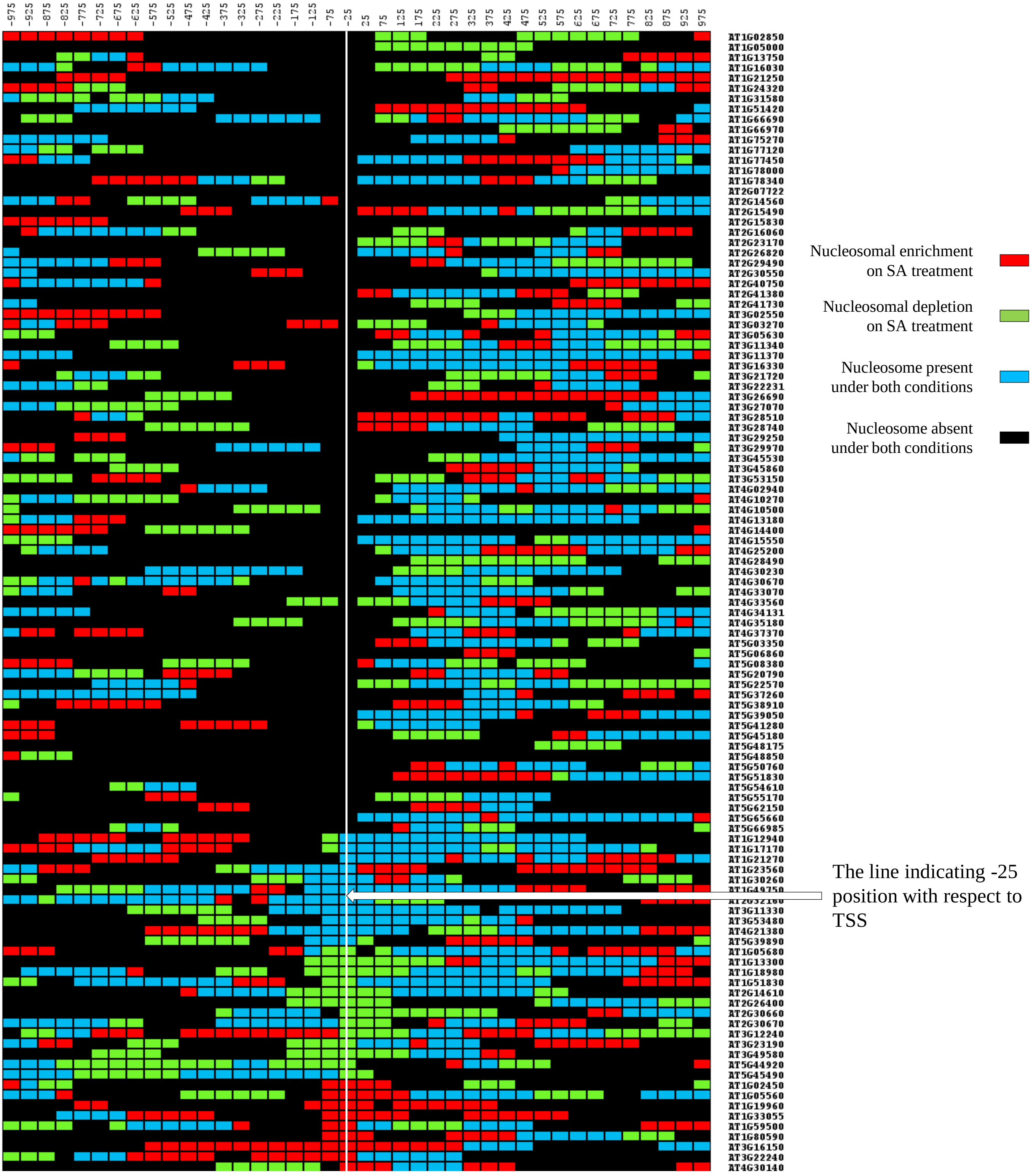

-1000bp to 1000bp region of all SI genes was mapped for the nucleosomal depletion, nucleosomal enrichment, Nucleosome present under both conditions, Nucleosome absent under both conditions

Additional figure 5: NPR1 plays a distinct role in the nucleosomal remodeling around the core promoter region of the Salicylic acid regulated genes

A

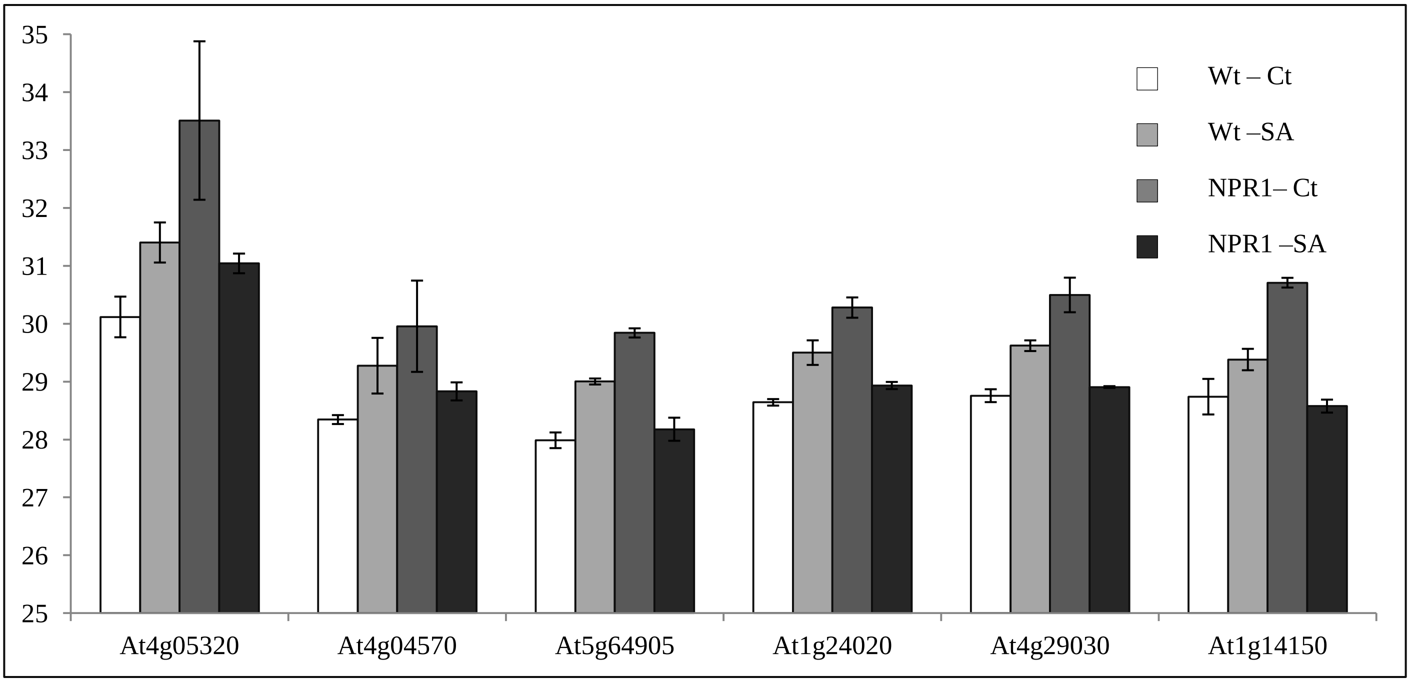

B

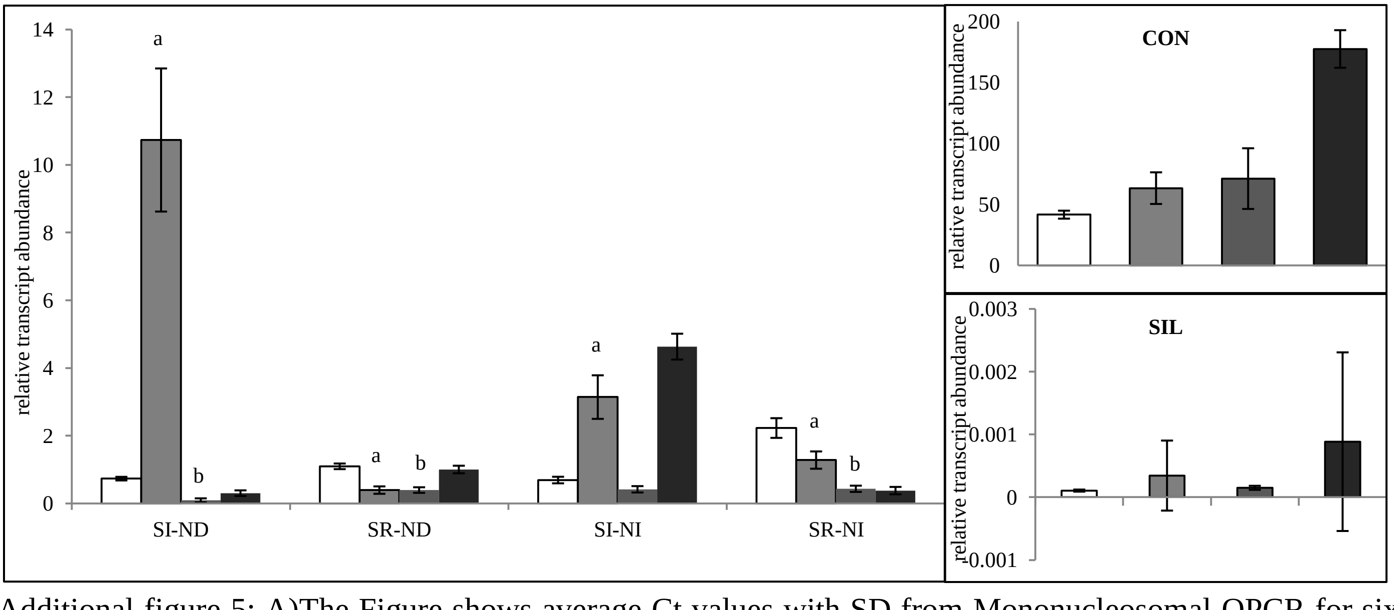

Additional figure 5: A)The Figure shows average Ct values with SD from Mononucleosomal QPCR for six different genes having constant nucleosomal occupancy regardless of treatments or presence of NPR1 (cwn- mononucleosomal DNA from untreated whole seedlings of col-0 ecotype, csn- mononucleosomal DNA from Salicylic acid (SA) treated whole seedlings of col-0 ecotype, nwn- mononucleosomal DNA from untreated whole seedlings of *npr1* mutant, nsn- mononucleosomal DNA from SA treated whole seedlings of *npr1* mutant). B) The figure shows average relative transcript abundance in wildtype (Wt) and *npr1-1* mutants(NPR1) under untreated (Ct) and treated (SA) conditions of three genes from each group after normalization with a set of five internal control genes and with Standard Error (SE). 'a' is significant change in value obtained when Student's t-test was applied for treated samples at $p \leq 0.05$ against the untreated counterparts. 'b' denotes the significant change in value when Student's t-test was applied at $p \leq 0.05$ for *npr1-1* mutant against Col-0 ecotype for respective treatments. For better visibility constitutive (CON) and silent (SIL) graphs are plotted separately.
